# Chemokines: structure, receptors and functions. A new target for inflammation and asthma therapy?

**DOI:** 10.1155/S0962935196000567

**Published:** 1996-12

**Authors:** F. A. A. van Acker, H-P. Voss, H. Timmerman

**Affiliations:** 1 Leiden/Amsterdam Center for Drug Research (LACDR) Department of Pharmacochemistry, Faculty of Chemistry Vrije Universiteit De Boelelaan 1083 Amsterdam 1081 HV The Netherlands

## Abstract

Five to 10% of the human population have a disorder of the respiratory tract called ‘asthma’. It has been known as a potentially dangerous disease for over 2000 years, as it was already described by Hippocrates and recognized as a disease entity by Egyptian and Hebrew physicians. At the beginning of this decade, there has been a fundamental change in asthma management. The emphasis has shifted from symptom relief with bronchodilator therapies (e.g. β_2_-agonists) to a much earlier introduction of anti-inflammatory treatment (e.g. corticosteroids). Asthma is now recognized to be a chronic inflammatory disease of the airways, involving various inflammatory cells and their mediators. Although asthma has been the subject of many investigations, the exact role of the different inflammatory cells has not been elucidated completely. Many suggestions have been made and several cells have been implicated in the pathogenesis of asthma, such as the eosinophils, the mast cells, the basophils and the lymphocytes. To date, however, the relative importance of these cells is not completely understood. The cell type predominantly found in the asthmatic lung is the eosinophil and the recruitment of these eosinophils can be seen as a characteristic of asthma. In recent years much attention is given to the role of the newly identified chemokines in asthma pathology. Chemokines are structurally and functionally related 8–10 kDa peptides that are the products of distinct genes clustered on human chromosomes 4 and 17 and can be found at sites of inflammation. They form a superfamily of proinflammatory mediators that promote the recruitment of various kinds of leukocytes and lymphocytes. The chemokine superfamily can be divided into three subgroups based on overall sequence homology. Although the chemokines have highly conserved amino acid sequences, each of the chemokines binds to and induces the chemotaxis of particular classes of white blood cells. Certain chemokines stimulate the recruitment of multiple cell types including monocytes, lymphocytes, basophils, and eosinophils, which are important cells in asthma. Intervention in this process, by the development of chemokine antagonists, might be the key to new therapy. In this review we present an overview of recent developments in the field of chemokines and their role in inflammations as reported in literature.


Mediators of Inflammation, 5, 393–416 (1996)

### Chemokines: structure, receptors and functions. A new target for inflammation and asthma therapy?

F. A. A. van Acker,[CA] H-P. Voss and H. Timmerman

Leiden/Amsterdam Center for Drug Research (LACDR), Department of Pharmacochemistry, Faculty of Chemistry, Vrije Universiteit, De Boelelaan 1083, 1081 HV Amsterdam, The Netherlands

[CA]Corresponding Author
Fax: (+31) 20 444 7610

**FIVE to 10% of the human population have a disorder of the respiratory tract called 'asthma'. It has been known as a potentially dangerous disease for over 2000 years, as it was already described by Hippocrates and recognized as a disease entity by Egyptian and Hebrew physicians. At the beginning of this decade, there has been a fundamental change in asthma management. The emphasis has shifted from symptom relief with bronchodilator therapies (e.g. $\beta_2$-agonists) to a much earlier introduction of anti-inflammatory treatment (e.g. corticosteroids). Asthma is now recognized to be a chronic inflammatory disease of the airways, involving various inflammatory cells and their mediators. Although asthma has been the subject of many investigations, the exact role of the different inflammatory cells has not been elucidated completely. Many suggestions have been made and several cells have been implicated in the pathogenesis of asthma, such as the eosinophils, the mast cells, the basophils and the lymphocytes. To date, however, the relative importance of these cells is not completely understood. The cell type predominantly found in the asthmatic lung is the eosinophil and the recruitment of these eosinophils can be seen as a characteristic of asthma. In recent years much attention is given to the role of the newly identified chemokines in asthma pathology. Chemokines are structurally and functionally related 8–10 kDa peptides that are the products of distinct genes clustered on human chromosomes 4 and 17 and can be found at sites of inflammation. They form a superfamily of proinflammatory mediators that promote the recruitment of various kinds of leukocytes and lymphocytes. The chemokine superfamily can be divided into three subgroups based on overall sequence homology. Although the chemokines have highly conserved amino acid sequences, each of the chemokines binds to and induces the chemotaxis of particular classes of white blood cells. Certain chemokines stimulate the recruitment of multiple cell types including monocytes, lymphocytes, basophils, and eosinophils, which are important cells in asthma. Intervention in this process, by the development of chemokine antagonists, might be the key to new therapy. In this review we present an overview of recent developments in the field of chemokines and their role in inflammations as reported in literature.**

**Key words:** Airway inflammations, Asthma, Chemokines

### Introduction

Diseases characterized by airway inflammation, excessive airway secretion and airway obstruction affect a substantial proportion of the population. These diseases include asthma, chronic bronchitis, bronchiectasis and cystic fibrosis.

Asthma has been the subject of extensive research for many years. This is not surprising as asthma is a frequently occurring disease with a history of a high morbidity and mortality. Until

© 1996 Rapid Science Publishers a few years ago the primary symptoms of asthma were thought to be increased airway responsiveness and recurrent 'reversible' airway obstruction. This is shown by the definition of asthma by the American Thoracic Society dating from 1987.

> Asthma is a clinical syndrome characterised by increased responsiveness of the tracheo-bronchial tree to a variety of stimuli. The major symptoms of asthma are attacks of dyspnea (disorder of breathing), wheezing and cough, which may vary from mild and almost undetectable to severe and unremitting (status asthmaticus). The primary physiological manifestation of this hyperresponsiveness is variable airway obstruction. This can take the form of spontaneous fluctuations in the severity of obstruction, substantial improvements in the severity of obstruction following bronchodilators or corticosteroids, or increased obstruction caused by drugs or other stimuli . . ..[1]

The major goal in treatment was to reverse this airway obstruction.

At the beginning of this decade, a fundamental change in asthma management took place. The emphasis has shifted from symptom relief with bronchodilator therapies to a much earlier introduction of anti-inflammatory treatments.[2] Based on a growing body of evidence, allergic as well as intrinsic bronchial asthma have been defined as chronic persistent inflammatory disorders. Agreement has been reached that asthma can no longer be seen as an equivalent of bronchospasm and that the absence of reversibility of airflow obstruction does not exclude bronchial asthma.[3]

Thus, asthma is now recognized to be a chronic inflammatory disease of the airways, involving amongst others mast cells, eosinophils and T-lymphocytes. Airway production of chemokines, cytokines and growth factors in response to irritants, infectious agents and inflammatory mediators also play an important role in the modulation of acute and chronic airway inflammation.[4] Treatment of asthma should therefore be based on anti-inflammatory agents rather than bronchodilators.[2]

The asthmatic attack can be divided in two main phases: the immediate- or early-phase asthmatic response and the delayed- or late-phase reaction. This division is fairly arbitrary, because in some subjects only one of the phases may be obvious, but it provides a useful basis for discussing the physiopathological changes in the bronchi and the mediators that are involved.[5] The early-phase, i.e. the initial response, occurs abruptly and is due mainly to spasm of the bronchial smooth muscle. After a challenge with all kinds of stimuli, the alveolar macrophages will be activated and produce mediators. Other primary effector cells, such as mast cells and epithelium cells also contribute to the production of mediators. Some of these mediators, e.g. histamine, $LTC_4$, $LTD_4$ and prostaglandin $D_2$ will cause direct contraction of the airway smooth muscle, producing a bronchospasm and thus an airway obstruction. Various chemotaxins (e.g. $LTB_4$ and chemokines) initiate the inflammatory reaction in the airways by attracting leukocytes into the area and hence preparing for the late-phase reaction.

The second, late-phase occurs in approximately 50% of the asthmatics (even more in children)[6] at a variable time after exposure to the elicting stimulus. This phase is in essence a progressing inflammatory reaction and is caused by the infiltration of, amongst others, airway obstruction neutrophils and eosinophils. Eosinophils especially play an important role in the pathogenesis of asthma.[7] Most of the products released by these cells have been tested for their effects on lung tissue. They all have some effects (extensively reviewed by Barnes[8]) but none of them is solely responsible for the observed phenomenon in the asthmatic reaction.[9]

### Inflammatory Cells

A number of studies have provided information on cell populations in bronchoalveolar lavage (BAL) fluid in mild, stable asthmatics with persistent airways hyperresponsiveness and asthma.[10–12] Common findings in these studies, as well as in recent examinations of bronchial mucosal biopsies,[13,14] are the presence of increased numbers of inflammatory cells, such as eosinophils, lymphocytes and mast cells, compared with normal control subjects with normal airway responsiveness. The eosinophils have shown signs of activation, as indicated by increased levels of granular proteins, major basic protein (MBP) and eosinophilic cationic protein (ECP).[15] Both MCP and ECP are cytotoxic for airway epithelium.[1] Azzawi *et al.*[16] have also demonstrated significant increases in the number of activated T-lymphocytes. Mast cells in the airways mucosa have exhibited various stages of degranulation,[14] suggesting that mediator release is an ongoing process in the airways of stable asthmatics with persistent airway hyperresponsiveness. These inflammatory cells release a wide variety of mediators, including local release of preformed mediators, newly synthesized metabolites of arachidonic acid, and soluble pro-inflammatory proteins including kinins and cytokines.[17] Airway epithelial cells participate in local cytokine networks and regulate inflammatory airway events by synthesizing and secreting various cytokines that communicate in a paracrine manner with infiltrating inflammatory cells and structural airway cells. Furthermore, airway epithelial cells represent targets for numerous cytokines that regulate the expression of immune and inflammatory airway epithelial cell products.

### Mediators

Release of inflammatory mediators such as histamine and products of arachidonic acid metabolism has been demonstrated in BAL fluid of patients with asthma. Airway inflammation in asthma is a complex series of events triggered by inflammatory stimuli interacting with primary effector cells resident within the airways. Release of inflammatory mediators from these cells may in turn recruit and activate other effector cells or cell-independent systems, with generation of other mediators, thus augmenting the inflammatory process.[8] These include preformed mediators, such as histamine, mediators newly synthesized by basophils or mast cells after antigen stimulation, such as leukotrienes, and mediators generated secondarily as a result of primary mediator release. An example of the latter is bradykinin, which is generated by the action of kallikrein on serum kininogen. Still other mediators are released from actively recruited cells over longer periods of time (e.g. eosinophil granule constituents, cytokines, chemokines), and their importance in the immunopathogenesis of asthma has been inferred based on their detection within the asthmatic airway, or following experimental allergen challenge.[17] Inflammatory mediators may have a variety of effects on several target cells within the airway and may mimic many of the features found in asthma. They may lead to contraction of the airway smooth muscle, either directly or indirectly, through the release of other mediators, or the activation of neural pathway.[8]

### Preformed mediators

Histamine was the first inflammatory mediator studied, having been synthesized in 1907 and studied extensively by Dale and Laidlaw in the years thereafter.[18] Histamine is generated in basophils and mast cells by the enzymatic decarboxylation of histidine. Elevations of histamine in BAL fluids have been found in the airways of asthmatics, and the levels increase strikingly within minutes and even many hours following antigen challenge. The other preformed mediators in human basophils and mast cells have as yet no well defined roles in the pathophysiology of asthma. Human mast cells contain tryptase, chymase, carboxypeptidase A, and several acid hydrolases, while basophils contain lysophospholipase.[17]

### Newly synthesized mediators

The non-preformed mediators derived from basophils, eosinophils, mast cells, and other sources are also known as the lipid mediators. With the exception of platelet activating factor (PAF), they are products of arachidonic acid metabolism through two different pathways. The cyclooxygenase pathway is responsible for the generation of prostaglandins, prostacyclin, and thromboxane, while the lipoxygenase pathway generates leukotrienes and HETES (hydroxyeicosatetraenoic acids). In the 5-lipoxygenase pathway, arachidonic acid undergoes lipoxygenation to produce leukotriene $A_4$ ($LTA_4$). This is subsequently metabolized to $LTB_4$ or $LTC_4$. $LTC_4$ in turn is rapidly metabolized to $LTD_4$ and $LTE_4$. In physiologic studies the leukotrienes seem to have about the same range of activities as does histamine, and levels of leukotrienes within the airways are higher in asthma. The cyclooxygenase enzyme catalyzes the incorporation of molecular oxygen into the arachidonic acid and promotes ring closure to form the relatively unstable cyclic endoperoxides $PGG_2$ and $PGH_2$. These are converted to the primary prostaglandins such as $PGD_2$, $PGE_2$, and $PGF_{2\alpha}$. Alternatively, the endoperoxides may also be metabolized to prostacyclin ($PGI_2$) or thromboxane $A_2$. Prostaglandin $D_2$ is the predominant prostanoid generated by mast cells; none is generated in human basophils.[19] A cyclooxygenase subtype, cyclooxygenase-2, is induced during inflammation. Therefore, the prostaglandin production will be increased during inflammatory processes.

Until recently, PAF was thought to be one of the most important mediators in the pathogenesis of asthma. This was because it mimics many features of asthma and, in addition to having physiologic activities much like those of histamine, it also is chemotactic for eosinophils and other inflammatory cells *in vivo*.[20] However, it has subsequently been found that other mediators such as the leukotrienes may have a similar activity.[17]

Other mediators generated subsequent to mast cell and basophil mediator release are the kinins. Bradykinin has effects similar to the tachykinins (neurokinins A and B, substance P). When inhaled, it is a potent bronchoconstrictor and causes a sensation of dyspnoea similar to asthma. This is probably due to an action on sensory nerves within the airways. Levels of kinins have been found to be elevated in asthmatic airways and to increase even further after segmental antigen challenge.[17]

### Other pro-inflammatory proteins

A host of cytokines released by T-lymphocytes and other cells are pivotal in mediating many inflammatory responses in allergic diseases including asthma. Detectable levels of mRNA for TNF, IL-1, IL-3, IL-4, IL-5, and GM-CSF has been reported in biopsies or BAL fluids. A similar but slightly different panel of cytokine proteins has also been observed (e.g. IL-2 and IL-6 have also been detected). The source of these and other cytokines may include not only T-lymphocytes but also macrophages, epithelial cells, mast cells, basophils, and eosinophils.

As mentioned above, eosinophilic inflammation is a consistent and prominent finding in asthma. The eosinophil granule proteins (a second category of pro-inflammatory proteins), such as eosinophil cationic protein (ECP), eosinophil peroxidase (EPO), and major basic protein (MBP), are highly toxic to epithelium and other pulmonary cells, and inhalation of MBP can induce hyperreactivity.[21]

A third category of pro-inflammatory proteins are the chemokines, a special type of cytokines. In the remaining sections of this review the chemokines will be discussed.

### Chemokines and Inflammation

Chemokines are structurally and functionally related 8–10 kDa peptides that are the products of distinct genes clustered on human chromosomes 4 and 17. They are a superfamily of proinflammatory mediators that promote the recruitment of various kinds of leukocytes and lymphocytes.[22] Chemokines are strongly implicated in a wide range of human acute and chronic inflammatory diseases, including arthritis, respiratory diseases, and arteriosclerosis.[23] Additionally, they may play an important role in host defense against infections and in wound healing.

The invasion of the body by pathogenic organisms triggers a cellular response by the immune system that leads to the recruitment of leukocytes. The initial migration of leukocytes toward the site of infection (chemotaxis) is mediated by a variety of molecules, called chemoattractants or chemotaxins.[24] The chemoattractant is the signal that triggers a complex sequence of events dependent on interactions between adhesion molecules and their complementary ligands on leukocytes.[25]

Much of the knowledge concerning leukocyte chemoattractants originates from use of the Boyden chamber which measures chemotaxins *in vitro*. The invention of the chemotaxis chamber by Boyden in 1962 allowed *in vitro* quantification of leukocyte movements in defined gradients or soluble chemoattractants.[26] The first chemoattractant for neutrophils demonstrated using this system was the complement fragment C5a. By 1986, the structural and functional properties of the 'classical' chemoattractants *N*-formyl-methionyl-leucyl-phenylalanine (fMLF), C5a, leukotriene B4, and platelet-activating factor (PAF) had been extensively detailed.[27]

Recently, the number of structurally defined chemoattractants for leukocytes has greatly increased, largely due to the identification of the chemokine superfamily.[28] The name 'chemokine' was proposed at the Third International Symposium of Chemotactic Cytokines at Baden in 1992. 'Chemokine' combines the ***chemo***attractant and cyto***kine*** properties that have been identified for many of these peptides. Previous to this symposium the chemokines were termed 'intercrines'. Immunologists first detected a member of the intercrine family when Luster *et al.*[29] in 1985 reported the induction of gene expression for a peptide homologous to platelet proteins in interferon gamma (IFN-γ) stimulated macrophages and termed the peptide 'IP-10'. Subsequently, Yoshimura *et al.*[30,31] isolated and identified a novel monocyte cell-derived neutrophil chemoattractant, and they were the first to separate this peptide biochemically from IL-1 and TNF, which were previously considered to be responsible for this activity. This novel chemoattractant polypeptide was initially named 'monocyte-derived neutrophil chemotactic factor' (MDNCF). Various investigators have referred to this peptide as a 'neutrophil activating protein' (NAP), MDNCF, NAF, GCF, LCF, LAI and most recently IL-8.[28] The fact that the chemokines have remarkably conserved sequences, distinguishes them from the other chemoattractants and most other cytokines.[27]

Although the superfamily is defined by structure, three common functional properties are also apparent. Firstly, chemokines attract one or more myeloid cell types *in vitro*. Secondly, the production and/or secretion of most chemokines in source cells is induced by pro-inflammatory stimuli such as lipopolysaccharide, tumour necrosis factor-1 (TNF-1) or interleukin-1 (IL-1). Thirdly, all those chemokines that have been tested induce inflammatory infiltrates when injected intradermally into animals, although certain species barriers may exist.[32]

The human chemokine polypeptides are 70–90 residues in length and have internal disulphide bonds, comparable with C3a, C4a, and C5a. However, the chemokine and complement fragment sequences are only 15% identical. All chemokines have four cysteine residues which form two disulphide bridges.[28] Traditionally, the chemokine superfamily has been divided into two subgroups: CXC (α; C is cysteine and X is any amino acid) and CC (β), based on the chromosomal location of the gene, the overall sequence homology and the disposition of the first two of the four conserved cysteine residues (Fig. 1). All known α chemokines are 25–90% identical, while all known β chemokines are 25–70% identical. Any α chemokine is 20–30% identical to any β chemokine.

Recently, the discovery of a new protein suggests that the superfamily may have an additional branch, the 'C' (γ) branch. Lymphotactin, a molecule isolated from pro-T cells, clearly lacks the first and third cysteines in the four cysteine pattern, but shares a large amount of amino acid similarity at its carboxyl terminus with CC chemokines (Fig. 1).[33] The structural analysis, chromosomal location and biological properties of lymphotactin provide strong evidence that this cytokine represents a new class of chemokine.[34,35]

Although the chemokines have highly conserved amino acid sequences, each of the chemokines binds to and induces the chemotaxis of a particular class of white blood cells. CXC (α) chemokines (such as IL-8 and MGSA) stimulate predominantly neutrophils, except for platelet factor 4 (PF-4) and γ-interferon inducible protein (γIP-10). CC (β) chemokines (such as MIP-1α, MCP-1 and RANTES) on the other hand, do not affect neutrophils but stimulate multiple cell types including monocytes, lymphocytes, basophils, and eosinophils.[36] The C (γ) chemokine lymphotactin mainly attracts lymphocytes.

There are probably more structural distinctions to be made, which may explain/enlighten chemokine function. Within the CXC group, the majority of the known proteins contain the amino acid motif Glu-Leu-Arg-Cys-Xaa-Cys (ELRCXC or ELR) at the amino terminal region. These amino acids are absent in certain mem-

**CXC Chemokines**

```

IL-8          SAKELRCQCIKTYSKPFHPKFIKELRVIESGPHCANTEIIVKLSD.GRELCLDPKENWVQRVVEKFLKRAENS

NAP-2           AELRCMCIKTTSG.IHPKNIQSLEVIGKGTHCNQVEVIATLKD.GRKICLDPDAPRIKKIVQKKLAGDESAD

ENA-78   AGPAAAVLRELRCVCLQTTQG.VHPKMISNLQVFAIGPQCSKVEVVASLKN.GKEICLDPEAPFLKKVIQKILDGGNKEN

GRO-a         ASVATELRCQCLQTLQG.IHPKNIQSVNVKSPGPHCAQTEVIATLKN.GRKACLNPASPIVKKIIEKMLNSDKSN

GRO-b         APLATELRCQCLQTLQG.IHLKNIQSVKVKSPGPHCAQTEVIATLKN.GQKACLNPASPMVKKIIEKMLKNGKSN

GRO-c         ASVVTELRCQCLQTLQG.IHLKNIQSVNVRSPGPHCAQTEVIATLKN.GKKACLNPASPMVQKIIEKILNKGSTN

PF4          EAEEDGDLQCLCVKTTSQ.VRPRHITSLEVIKAGPHCPTAQLIATLKN.GRKICLDLQAPLYKKIIKKLLES

IP-10         VPLSRTVRCTCISISNQPVNPRSLEKLEIIPASQFCPRVEIIATMKKKGEKRCLNPESKAIKNLLKAVSKEMSKRSP

MIG           TPVVRKGRCSCISTNQGTIHLQSLKDLKQFAPSPSCEKIEIIATLKN.GVQTCLNPDSADVKELIKKWEKQVSQ

```

**CC Chemokines**

```
RANTES   SPYSSDT.TPC.CFAYIARPLPRAHIKEYFYTSGK..CSNPAVVFVTRKN.RQVCANPEKKWVREYINSLEMS
I309     SKSMQVPFSRC.CFSFAEQEIPLRAILCYRNTSSI..CSNEGLIFKLKRG.KEACALDTVGWVQRHRKMLRHCPSKRK
MIP-1α   ASLAADTPTAC.CFSYTSRQIPQNFIADYFETSSQ..CSKPGVIFLTKRS.RQVCADPSEEWVQKYVSDLELSA
MIP-1β   APMGSDPPTAC.CFSYTARKLPRNFVVDYYETSSL..CSQPAVVFQTKRS.KQVCADPSESWVQEYVYDLELN
MCP-1    QPDAINAPVTC.CYNFTNRKISVQRLASYRRITSSK.CPKEAVIFKTIVA.KEICADPKQKWVQDSMDHLDKQTQTPKT
MCP-2    QPDSVSIPITC.CFNVINRKIPIQRLESYTRITNIQ.CPKEAVIFKTKRG.KEVCADPKERWVRDSMKHLDQIFQNLKP
MCP-3    QPVGINTSTTC.CYRFINKKIPKQRLESYRRTTSSH.CPREAVIFKTKLD.KEICADPTQKWVQDFMKHLDKKTQTPKL
```

**C Chemokine**

```
LTN        GVEVSDKRT.CVSLTTQRLPVSRIKTYTITEG...SLR.AVIFITKRGLK.VCADPQATWVRDVVRSMDRKSNTRNNMIQT...
```

FIG. 1. Multiple sequence alignment of human CXC, CC and C chemokines.[68]

bers of the CXC chemokine family[37,38] in particular PF-4, γIP-10 and MIG (monokine inducible by gamma interferon). Recent investigations have demonstrated that the three amino acids preceding the first N-terminal cysteine (ELR) are critical for the neutrophil chemotactic and activating properties of these mediators.[25] Most of the ELR proteins are potent neutrophil chemoattractants, and have the capacity to bind a shared C-X-C chemokine receptor. In contrast, proteins lacking the ELR motif, have an altered chemotactic spectrum of activities and do not seem to bind the shared CXC receptor. Thus, in summary the chemokine superfamily may consist of at least four different structurally and functionally meaningful parts: (a) the CC subfamily (β); (b) the C subfamily (γ); (c) the CXC subfamily without ELR (non-ELR) (α); (d) the CXC subfamily with ELR (ELRCXC subfamily) (α). A schematic representation is depicted in Fig. 2.

### The Chemokine Receptors

The molecular target for chemokines are their cell surface receptors.[36] The chemotactic signals for leukocytes are transduced to heterotrimeric G proteins by receptors with seven predicted transmembrane domains. All of the chemokine receptors have seven domains enriched in hydrophobic amino acids, several of which are conserved among most members of the G-protein coupled receptor (GPCR) superfamily. There are no specific amino acids or amino acid patterns common to all chemoattractant receptors which can distinguish them from other types of GPCRs. Nevertheless, there are five general properties, which makes the chemoattractant receptors to a subfamily within the GPCR superfamily: (1) their sequences are similar in length, approximately 350 amino acids, (2) they have over 20% amino acid identity overall to each other, (3) the short third intracellular loop is enriched in basic amino acids. Many other GPCRs have long third intracellular loops, (4) the N-terminal segments are in most cases unusually acidic, and (5) their RNAs are expressed in leukocytes.[27]

### Signal transduction

The chemokine receptors are thought to regulate the activity of phospholipase C through activation of the G-protein. The activated G-protein causes a phospholipase C mediated breakdown of phosphatidylinositol-4,5-bisphosphate ($PIP_2$) to produce the second messengers inositol-1,4,5-trisphosphate (1,4,5-$IP_3$) and 1,2-diacylglycerol (DAG). $IP_3$ mobilizes intracellular calcium, while DAG activates protein kinase C (PKC).

### Receptor Subtypes

Based on binding specificity and expression in certain cell types, the chemokine receptors can be classified in different ways. According to

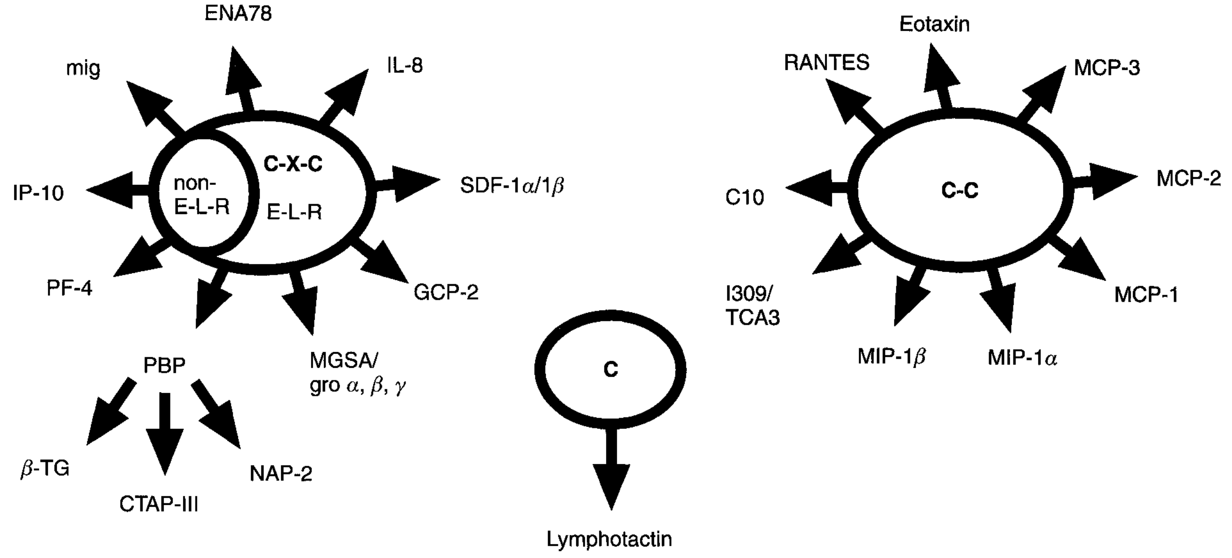

FIG. 2. Organization of the chemokine superfamily. Schematic depiction of a four-part superfamily structure, on the basis of the arrangement of amino acids around the conserved cyststeines in the proteins. The name used for each of the chemokines is that of the human protein, other names for homologues from other species may exist. The arrows originating from PBP indicate that three proteins with distinct activities (β-TG, CATP-III, and NAP-2) are proteolytically derived from that molecule.[39]

Schall and Bacon[39] the receptors can, so far, be grouped into four general classes.

### Promiscuous receptors

The promiscuous receptor is a receptor that binds chemokines of either CC or CXC classes. To date, the only example of this receptor is the erythrocyte chemokine receptor (ECKR). Horuk, Chaudhuri, and co-workers have shown that this erythrocyte chemokine binding protein is identical to the Duffy blood-group antigen, which is a receptor for the malarial parasite *Plasmodium vivax*.[40,41]

### Shared receptors

The shared receptor is a receptor which will bind to more than one chemokine within either the CXC or the CC class. Two examples are the interleukin-8 receptor B (IL-8B receptor) and the CC chemokine receptor-1 (CC CKR-1, also called the MIP-1α/RANTES receptor). The IL-8B receptor binds chemokines with the ELRCXC motif (CXC class), whereas the CC CKR-1 binds several of the CC chemokines.

### Specific receptors

These receptors seem to bind only one specific chemokine. The interleukin-8 receptor A (IL-8A receptor) and the monocyte chemoattractant protein (MCP)-1 receptor represent this class.

### Virally encoded receptors

To date there are two reports of virally encoded receptors. One is encoded by a cytomegalovirus open reading frame, CMV U28,[42,43] and the other from herpes saimiri virus, HSV ECRF3.[44] These two receptors are probably shared C-C and C-X-C receptors respectively, that have been transduced by viruses during evolutionay history.

Horuk[24] uses another classification. The chemokine receptors are divided into *CXC chemokine receptors, CC chemokine receptors, viral homologues of chemokine receptors* and the *human erythrocyte chemokine receptor*. The CXC chemokine receptors contain the IL-8A and IL-8B receptors. The CC chemokine receptors are represented by (1) a MIP-1α-MIP-1β-shared receptor, (2) a MCP-1-specific receptor, and (3) a MCP-1-MIP-1α-MIP-1β-shared receptor. Shall and Bacon[39] refer to the MIP-1α-MIP-1β-shared receptor as the MIP-1α/RANTES receptor, whereas the MCP-1-MIP-1α-MIP-1β-shared receptor is not taken into account. The class of the viral homologues of chemokine receptors and the human erythrocyte receptors represent the same receptors mentioned in the virally encoded receptors and promiscuous receptors by Schall and Bacon respectively.

### CXC Receptors

IL-8 is one of the best characterized CXC chemokines and selective receptors for IL-8 were demonstrated by several binding studies with human neutrophils. In general agreement with other reports,[45,46] Baggiolini and co-workers[47] found that human neutrophils possess on average 64 500 ± 14 000/cell receptors with an apparent $K_d$ of 0.18 ± 0.07 nM. Most of the studies on IL-8 receptors were initially carried out with [$^{125}$I]IL-8, since IL-8 was the first CXC chemokine that was available in sufficient quantity for receptor characterization.[24] Radiolabelled IL-8 is displaced by cold IL-8, but also by NAP-2 and GROα. The displacement of IL-8 by other CXC chemokines is bimodal, revealing the existence of at least two types of receptors on neutrophils, one with high affinity for all three ligands (IL-8RB; $K_d$ 0.1–0.3 nM), and the other with high affinity for IL-8, but low affinity for NAP-2 and GROα (IL-8RA; $K_d$ 100–130 nM).[45,46] Additionally, IL-8 is able to desensitize calcium transients elicited by GROα and NAP-2, but GROα and NAP-2 do not desensitize the response to IL-8.[27] The existence of two IL-8 receptors is further supported by the cloning of two cDNAs encoding seven-transmembrane-domain receptors, and causing binding of CXC chemokines to cells upon transfection.[47] These products have been referred to as IL-8 receptors A and B, B and A, α and β and Type 1 and Type 2 in literature, but in this review they will be termed A and B following the gene symbols published by Murphy.[27] The deduced sequences of the IL-8A receptor and the IL-8B receptor are highly homologous at the amino acid level (77%), whereas they are 23–30% homologous to other leukocyte chemoattractant receptors (Fig. 3).[27] IL-8A and IL-8B receptors have the highest homology over the membrane-spanning regions, and diverge at the amino and carboxyl termini.[48]

The IL-8RA (specific receptor which binds only IL-8) is more widely expressed than the IL-8RB (shared receptor) and is found on neutrophils as well as at low levels on monocytes and monocytic cell lines, melanoma cell lines, T cells, synovial fibroblasts, HL-60 and THP-1 myeloid precursor cell lines.[24,47] The expression of the IL-8RB receptor is more restricted and

```

           1

IL-8R-A    MESDSFEDFW KGEDLSNYSY SSTLPPFLLD AAPCEPESLE INKYFVVIIY

IL-8R-B    MSNITDPQMW DFDDLN.... FTGMPPADED YSPCMLETET LNKYVVIIAY

CC-CKR-1     METP.... ..NTTE.DYD TTTEFDYGDA TPCQKVNERA FGAQLLPPLY

CC-CKR-2    MLSTSRSRF IRNTNESGEE VTTFFDYDYG APCHKFDVKQ IGAQLLPPLY

CC-CKR-3     MTTS.... .LDTVE.TFG TTSYYD.DVG LLCEKADTRA LMAQFVPPLY

CC-CKR-4       MNPTDI ADTTLDESIY SNYYLYESIP KPCTKEGIKA FGELFLPPLY

                                                        <------

           51

IL-8RA     ALVFLLSLLG NSLVMLVILY SRVGRSVTDV YLLNLALADL LFALTLPIWA

IL-8RB     ALVFLLSLLG NSLVMLVILY SRVGRSVTDV YLLNLALADL LFALTLPIWA

CC-CKR-1   SLVFVIGLVG NILVVLVLVQ YKRLKNMTSI YLLNLAISDL LFLFTLPFWI

CC-CKR-2   SLVFIFGFVG NMLVVLILIN CKKLKCLTDI YLLNLAISDL LFLITLPIWA

CC-CKR-3   SLVFTVGLLG NVVVVMILIK YRRLRIMTNI YLLNLAISDL LFLVTLPFWI

CC-CKR-4   SLVFVFGLLG NSVVVLVLFK YKRLRSMTDV YLLNLAISDL LFVFSLPRWG

           ------ TM 1 ---------->         <--------- TM 2 ------

           101

IL-8RA     A.SKVNGWIF GTFLCKVVSL LKEVNFYSGI LLLACISVDR YLAIVHATRT

IL-8RB     A.SKVNGWIF GTFLCKVVSL LKEVNFYSGI LLLACISVDR YLAIVHATRT

CC-CKR-1   DYKLKDDWVF GDAMCKILSG FYYTGLYSEI FFIILLTIDR YLAIVHAVFA

CC-CKR-2   H SAANEWVF GNAMCKLFTG LYHIGYFGGI FFIILLTIDR YLAIVHAVFA

CC-CKR-3   HYVRGHNWVF GHGMCNLLSG FYHTGLYSEI FFIILLTIDR YLAIVHAVFA

CC-CKR-4   YY.AADQWVF GLGLCKMISW MYLVGFYSGI FFVMLMSIDR YLAIVHAVFS

           >                <---------- TM 3 ----------->

           151

IL-8RA     LTQKRY.LVK FICLSIWGLS LLLALPVLLF RRTVYSSNVS PACYEDMGNN

IL-8RB     LTQKRH.LVK FVCLGCWGLS MNLSLPFFLF TQAYHPNNSS PVCYEVLGND

CC-CKR-1   LRARTVTFGV ITSIIIWALA ILASMPGLYF SKTQWEFTHH TCSLHFPHES

CC-CKR-2   LKARTVTFGV VTSVITWLVA VFASVPGIIF TKCQKEDSVY VCGPYFP...

CC-CKR-3   LRARTVTFGV ITSIVTWGLA VLAALPEFIF YETEELFEET LCSALYPEDT

CC-CKR-4   LRARTLTYGV ITSLATWSVA VFASLPGFLF STCYTERNHT YCKTKYSLNS

                <------ TM 4 -------->

           201

IL-8RA     TANWRMLLRI LPQSFGFIVP LLIMLFCYGF TLRTLFKAHM GQK.HRAMRV

IL-8RB     TAKWRMVLRI LPHTFGFIVP LFVMLFCYGF TLRTLFKAHM GQK.HRAMRV

CC-CKR-1   LREWKLFQAL KLNLFGLVLP LLVMIICYTG IIKILLRRPN EKK.SKAVRL

CC-CKR-2   .RGWNNFHTI MRNILGLVLP LLIMVICYSG ILKTLLRCRN EKKRHRAVRV

CC-CKR-3   VYSWRHFHTL RMTIFCLVLP LLVMAICYTG IIKTLLRCPS KKK.YKAIRL

CC-CKR-4   T.TWKVLSSL EINILGLVIP LGIMLFCYSM IIRTLQHCKN EKK.NKAVKM

            <-------- TM 5 ---------->                         <

           251

IL-8RA     IFAVVLIFLL CWLPYNLVLL ADTLMRTQVI QETCERRNHI DRALDATEIL

IL-8RB     IFAVVLIFLL CWLPYNLVLL ADTLMRTQVI QETCERRNNI GRALDATEIL

CC-CKR-1   IFVIMIIFFL FWTPYNLTIL ISVFQDFLFT HE.CEQSRHL DLAVQVTEVI

CC-CKR-2   IFTIMIVYFL FWTPYNIVIL LNTFQEFFGL SN.CESTSQL DQATQVTETL

CC-CKR-3   IFVIMAVFFI FWTPYNVAIL LSSYQSILFG ND.CERSKHL DLVMLVTEVI

CC-CKR-4   IFAVVVLFLG FWTPYNIVLF LETLVELEVL QD.CTFERYL DYAIQATETL

           ---------- TM 6 ----------->                      <-----

           301

IL-8RA     GILHSCLNPL IYAFIGQKFR HGLLKILAIH .......... GLISKDSLPK

IL-8RB     GFLHSCLNPI IYAFIGQNFR HGFLKILAMH .......... GLVSKEFLAR

CC-CKR-1   AYTHCCVNPV IYAFVGERFR KYLRQLFHR. RVAVHLVKWL PFLSVDRLER

CC-CKR-2   GMTHCCINPI IYAFVGEKFR RYLSVFFRK. HITKRFCKQC PVFYRETVDG

CC-CKR-3   AYSHCCMNPV IYAFVGERFR KYLRHFFHR. HLLMHLGRYI PFLPSEKLER

CC-CKR-4   AFVHCCLNPI IYFFLGEKFR KYILQLFKTC RGLGVLCQYC GLLQIYSADT

           ------ TM 7 ------>

           351
IL-8RA     DSRPSFVGSS SGHTSTTL
IL-8RB     HRVTSY.TSS SVNVSSNL
CC-CKR-1   VSST.SPSTG EHELSIVF
CC-CKR-2   VTSTNTPSTG EQEVSAGL
CC-CKR-3   TSSV.SPSTA EPELSIVF
CC-CKR-4   PSSSYTQSTM DHDLHDAL
```

FIG. 3. Multiple protein sequence alignment of the human chemokine receptors.[68] The seven putative transmembrane sequences are indicated by arrows.

confined primarily to myeloid cells including neutrophils, HL-60, THP-1 and AML 193 cells.[24,27] This suggests that the reported ability of IL-8 to attract small numbers of T cells may be mediated by IL-8RA.[27]

### Regulation of the expression of the IL-8 receptor A/B

It has been reported that [$^{125}$I]IL-8 bound to the IL-8R on neutrophils is rapidly internalized and degraded in lysosomes.[49–51] More than 90% of the ligand-bound receptors are endocytosed within 10 min at 37°C, and the receptors are recycled, as indicated by their re-expression on the cell surface approximately 10 min later.[49] Inhibitory lysosomotropic agents (agents that show a special affinity for lysosomes), including ammonium chloride, inhibit the internalization process. Ammonium chloride also inhibits chemotaxis, suggesting that chemotaxis may require internalization and reexpression of the IL-8 receptor. Chuntharapai and Kim[52] investigated the rate of down-modulation of IL-8RA/B expression by IL-8 on neutrophils and found that regardless of the expression level of IL-8RA and IL-8RB among different blood donors,[53] the $EC_{50}$ of IL-8 required for the down-modulation of IL-8RA was higher than that of IL-8RB. It was found to be impossible to down-modulate IL-8RA/B completely and this is probably due to the two ongoing competitive processes: down-modulation of receptors by the agonist and reappearance of receptors after dissociation from the bound ligand.

Investigations concerning the recycling of the receptors revealed that after the exogenous IL-8 was removed, the level of IL-8RA continued to increase and reached 85% of the untreated fresh control level during a 1.5-h culture period. In contrast, the level of IL-8RB recovered to only ~40% of the control value during a 1-h culture period and then remained at that level. The rapid re-expression of IL-8RA, with respect to IL-8RB, after 'complete' down-modulation supports the hypothesis that IL-8RA may play a more active role in transmitting the IL-8 signal in the inflammatory area compared with IL-8RB.[52]

It has been generally accepted that IL-8RA/B have similarly high affinities for IL-8,[22,54,55] although the magnitudes of the affinities reported varied from 0.1 to 4 nM. It is striking, that Chuntharapai and Kim[52] have a different view, by stating that their results obtained from the comparison of the $EC_{50}$ of IL-8 and the $K_d$ of each receptor for IL-8 clearly demonstrate that IL-8RB has a higher affinity for IL-8 compared with IL-8RA. They detected seven- to 13-fold and two- to five-fold differences in the $EC_{50}$ of IL-8 and the $K_d$ values, respectively.

As a result, they proposed a mechanism that could occur during inflammation. In the course of inflammation, resident macrophages and fibroblasts, located at the site of inflammation, secrete IL-8, and this secreted IL-8 gradually reaches nearby blood vessels. At a distant site, the concentration of IL-8 could be in the picomolar range, and at these concentrations IL-8RB would receive the IL-8 signal first and initiate the migration of neutrophils toward the inflammatory area. As neutrophils migrate closer to the site of inflammation, the IL-8 concentration can increase to the nanomolar range. At these IL-8 concentrations, IL-8RA would be the major receptor involved in mediating the IL-8 signal, since few IL-8RB would remain on the cell membrane.[52] Thus, the different affinities of IL-8RA/B for IL-8 may result in a different function; the low affinity IL-8RA may play an active role in mediating IL-8 signal in the inflammatory area, while the high affinity IL-8RB may initiate the neutrophil migration in a distant area of infection.

### Amino acids important for ligand binding: location of the active site

IL-8 may be an important mediator in various inflammatory exudates, including synovial fluid from patients with rheumatoid arthritis[56] and sputum from patients with cystic fibrosis, chronic bronchitis, or bronchiectasis.[47] Small molecule antagonists of IL-8 may therefore have the potential to be powerful anti-inflammatory agents. In order to assist the rational design of such compounds, it is important to elucidate the structure/function relationships of IL-8 and its receptors.

It has been reported that the N-terminal region of IL-8 is critical for ligand binding to neutrophils.[57,58] In particular, the single point substitution of Arg6 by Ala or Lys causes a 1000-fold decrease in the affinity of the ligand for its receptor.[58] Mutation of other amino acids in this region do not lead to a similar decrease in binding affinity. As the guanidinium side chain of the Arg6 residue of IL-8 is positively charged and is known to be pointing away from the core of the molecule,[58] it is likely to be poised to directly interact with a negatively charged amino acid side chain exposed on the ligand-accessible surface of the IL-8 receptor. By site-directed mutagenesis with systematic substitution of all the acidic residues present on the surface of the type A IL-8 receptor, this key residue was identified.[56] In the GPCR family, the ligand accessible surface is defined as the combination of the extracellular domain and part of the transmembrane domain. It is interesting to note that Asp85, which is located in the second transmembrane domain of the receptor (Fig. 4), is conserved in more than 90% of the members of the GPCR superfamily and may be a key residue maintaining the tertiary structure and proper folding of the receptor.

Replacement of Glu275 or Arg280 from the receptor by Ala causes a complete loss of IL-8 receptor binding. Sequence alignment shows that these residues are strictly conserved in the two human (type A and B), the rabbit, and the mouse IL-8 receptors. This demonstrates that the third extracellular loop of the receptor, which includes these Glu275 and Arg280, is an important functional domain of the receptor. Although Glu275 appears to be critical for binding, there is no evidence that it is involved in a direct interaction with the Arg6 of IL-8. Hébert and co-workers[56] speculate that Glu275 and Arg280 interact with Arg6 and Glu4 of IL-8, respectively.

The presence of Asp11 in the receptor appears to be critical for IL-8 binding as well, but it can be substituted with another acidic residue, such as Glu, or with Lys (found at the equivalent position in the IL-8RB). The substitution with Lys suggests that either Lys11 recruits a new and favourable interaction with IL-8 (analogous to that of IL-8RB with IL-8) or that the cavity created by mutating Asp11 to Ala is particularly disadvantageous. Results of studies with chimeric receptors in which the N-terminal segments of IL-8RB and rabIL-8R or IL-8RB and IL-8RA are switched clearly implicate this domain in determining the selectivity of the receptors.[27,48] Moreover, because α chemokines, such as IL-8, are fairly basic proteins (pI of IL8: 8–8.5),[56] the highly acidic N-termini of the IL-8RA/B could be a major determinant for ligand binding.

Nearly all members of the GPCR superfamily have a pair of conserved cysteines in extracellular loops 1 and 2, which are thought to form a disulphide bridge linking these two loops[59] (Fig. 4). Human IL-8 receptor type A and B as well as rabbit and mouse IL-8 receptor each contain two additional cysteines: one in the N-terminal region and the other in the extracellular loop 3

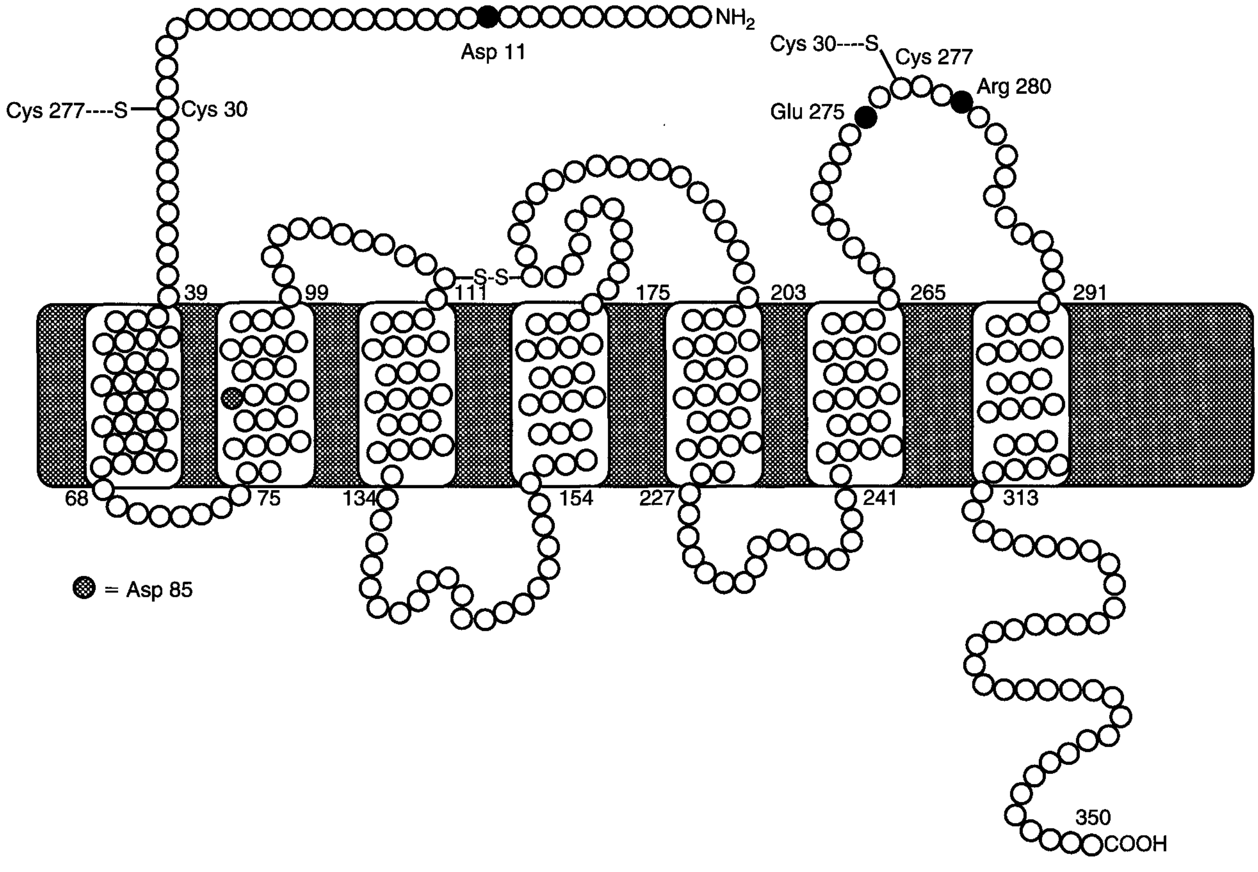

FIG. 4. Model for the secondary structure of IL-8 receptor type A. Residues that are critical for ligand binding are indicated in black. Asp 85 is conserved in more than 90% of the members of the GPCR superfamily and may be a key residue maintaining the tertiary structure and proper folding of the receptor.

(in the case of IL-8RA: Cys30 and Cys277). These two cysteines are very likely to interact with each other, forming a disulphide bridge which brings the N-terminal region and extracellular loop3 of the receptor in close spatial proximity. Hébert *et al.*[56] propose that Asp11, Glu275, and Arg280 of the IL-8 receptor type A are brought in close spatial proximity to each other by a disulphide bridge between Cys30 and Cys277 and constitute a major binding domain for IL-8. The binding domain of the IL-8RB receptor will be defined in a similar way.

### CC Chemokine Receptors

Over the past few years several new findings were published, which have significantly extended the knowledge about CC chemokine receptors. In 1993, direct binding data for the CC chemokines were sparse compared with that for the CXC chemokine receptors. A limited number of studies using radiolabelled MIP-1α and MIP-1β have been described. Interestingly these radiolabelled chemokines could be displaced by the CC chemokines MCP-1 and RANTES, but not by the CXC chemokines IL-8 and MIP-2. All four of these CC chemokines (MIP-1α/B, MCP-1, and RANTES) stimulated monocytes to carry out a variety of functions, and MIP-1α, MIP-1β, and RANTES had also been shown to stimulate chemotaxis and adhesion of T cells.[48] In addition, the members of this group attracted and activated polymorphonuclear leukocytes (PMN), eosinophils and lymphocytes with variable selectivity and MIP-1α had been shown to regulate the proliferative capacity of myeloid progenitor cells.[60]

Competitive inhibition studies using various CC chemokine ligands demonstrated that these chemokines and their receptors exhibited promiscuity similar to that of the CXC chemokine subfamily and the IL-8 receptors. MCP-1 binding could be partially displaced by either MIP-1α or MIP-1β. MIP-1α binding could be completely displaced by MIP-1β, and vice versa, and both were partially (30%) displaced by MCP-1.[61] These results suggested that at least three types of CC receptors were expressed on monocytic cells: (1) a specific receptor for MCP-1, (2) a shared receptor for MIP-1α and MIP-1β, which binds both ligands with equal affinity, and (3) a shared receptor for MIP-1α, MIP-1β and MCP-1. Additional studies with [$^{125}$I]RANTES indicated that RANTES bound with an affinity of 400–600 pM to monocytes and expressed approximately 600 receptors per cell.[62] RANTES binding could be completely displaced by MCP-1, MIP-1α and β. The affinities of these ligands for the receptor, however, are lower than for RANTES, which indicates that the third promiscuous receptor mentioned was perhaps the primary RANTES receptor, even though this receptor could accommodate also the other β ligands.[48] This promiscuous receptor was called the MIP-1α receptor,[24] the MIP-1α/RANTES receptor or the CC CKR1, the high affinity for RANTES has however not been found in the study of Neote *et al.*[42]

In 1993 only one gene had been reported for a leukocyte CC chemokine receptor, the human MIP-1α/RANTES receptor.[27] The receptor belongs to the GPCR superfamily, and its amino acid sequence showed approximately 32% identity with the IL-8A and IL-8B receptors, but only approximately 23% identity with the C5a and fMet-Leu-Phe (classical chemoattractant) receptors. Thus MIP-1α, MIP-1β, MCP-1 and RANTES all bind to the CC CKR1 with varying affinities and all four ligands can cross-compete for binding.[24] Chemokine binding affinity does not predict how well the ligand will transmit a signal through the receptor: RANTES and MIP-1α induce a similar intracellular calcium flux (at concentrations of 10–100 nM) while binding with disparate affinities, whereas MCP-1 and MIP-1β induce calcium mobilization only at high concentrations (20% of the RANTES/MIP-1α response at 1 μM).

Since 1993 new information became available concerning these receptors and in 1995 cDNAs for four human leukocyte CC chemokine receptors have been cloned. These receptors are designated CC CKR1, CC CKR2A and CC CKR2B (a single gene that produces two splice variants that differ in their carboxy terminal domains,[63] also known as MCP-1 receptors A and B) and CC CKR3. The properties of the first three receptors are not fully consistent with eosinophil chemotactic responses to CC chemokines.[64] MIP-1α and RANTES are efffective agonists for CC CKR1; however, its RNA is scarce in eosinophils. Much higher expression is found in neutrophils, monocytes and lymphocytes.[64] MCP-1 is an agonist for CC CKR2A and -2B, but it does not activate eosinophils. Moreover, CC CKR2 RNA is expressed in monocytes but not in eosinophils.[65] CC CKR3 is the first eosinophil-selective member of this family.

The CC CKR3 cDNA is 1.6 kb in length and it encodes a predicted protein of 355 amino acids that is identical in length and 63% identical in sequence with CC CKR1. CC CKR3 has 51% identity with CC CKR2B but only 31% identity with the CXC chemokine receptor and IL-8 receptors A and B (Fig. 3). The amino acid positions that differ between CC CKR1 and CC

CKR3 are found mostly in the (putative) extracellular domains and adjacent portions of the transmembrane domains. Like CC CKR1 and all other known chemokine receptors, the CC CKR3 sequence is acidic in the N-terminal segment before the first putative transmembrane domain. The second extracellular loop is also highly acidic, whereas for CC CKR1 the corresponding region is basic. In agreement with all other known chemokine receptors, CC CKR3 has conserved cysteine residues in the N-terminal segment and the third predicted extracellular loop that could form a disulphide bond.[64]

### Distribution of CC CKR3 RNA

The mRNA encoding the CC CKR3 receptor was first established in human peripheral blood-derived eosinophils and in small amounts in neutrophil and monocyte samples. Combadiere *et al.*[64] however, detected CC CKR1 mRNA in large amounts in neutrophil and monocyte samples and trace amounts in eosinophils. mRNA for CC CKR2B was found only in monocyte samples. Thus, CC CKR1, -2 and -3 are differently expressed in a cell type-specific pattern in human peripheral blood leukocytes.

Since MIP-1α, RANTES, and MCP-3 are the only known human CC chemokines that activate eosinophils, they were the best candidate agonists for CC CKR3. However, when three independent human embryonic kidney (HEK) 293 cell clones stably transfected with CC CKR3 were tested, all three exhibited $[Ca^{2+}]$ transients in response to MIP-1α, RANTES, and MIP-1β but not in response to MCP-1, MCP-2, MCP-3, IL-8 or γIP-10. The rank order of potency was MIP-1α > RANTES > MIP-1β. As previously reported, HEK 293 cells stably transfected with CC CKR1 also responded to MIP-1α and RANTES.[42] However, unlike CC CKR3, CC CKR1 transfected cells also responded to MCP-3 but not to MIP-1β at 100 nM. Since MIP-1α, RANTES, and MIP-1β are agonists for CC CKR3, they must bind to it. Nevertheless, Combadiere and colleagues[64] have not yet been able to demonstrate specific binding of $[^{125}I]$MIP-1α and $[^{125}I]$RANTES to CC CKR3-transfected HEK 293 cell using as much as 0.5 nM radioligand on 2 million transfected cells. This suggests that MIP-1α, MIP-1β and RANTES activate CC CKR3 via low binding interactions. In 1995 it was reported that it may well be that CC CKR3 is more selective for another, as yet untested, CC chemokine such as eotaxin.[66] Human eotaxin was not yet identified at that time. In January 1996, it was reported that this receptor indeed functions in response to eotaxin.[67] The studies strongly suggest that normal human monocytes and eosinophils respond to MIP-1α and RANTES via two MIP-1α/RANTES receptors, CC CKR1 and CC CKR3. The relative RNA distributions suggest that CC CKR1 functions principally, but not exclusively, in monocytes, and CC CKR3 functions principally, but not exclusively, in eosinophils.

Only very recently, Wells *et al.*[68] reported the identification of a fourth CC chemokine receptor in the human basophilic cell line KU-812. They have called it K5.5, or CC CKR4 and this receptor shows 49% identity with CC CKR1 over 356 amino acids, 46% identity to the CC CKR2 (form B) over 360 amino acids, and 45% with CC CKR3 over 356 amino acids. Northern blot analysis showed high levels of expression of CC CKR4 in the thymus and in peripheral blood leukocytes. They also showed that CC CKR4 was specifically expressed in T-cells, B-cells, and monocytes, as well as in platelets. Human basophils showed barely detectable CC CKR4 expression. However, after stimulation for 15 min with IL-5 (10 ng/ml) there was a significant up-regulation of receptor mRNA expression. The ligands for CC CKR4 were initially determined to be MCP-1, MIP-1α, and RANTES from measurements of $Ca^{2+}$-activated chloride currents in *Xenopus laevis* oocytes injected with cRNA for CC CKR4. The results for MIP-1α and RANTES have been confirmed by binding experiments using transfected cell lines.[68]

### The Erythrocyte Receptor

Erythrocytes have long been appreciated as transporters and exchangers of $O_2$ and $CO_2$ between the lungs and tissues. The observation that IL-8 can bind to erythrocytes in a saturable manner, suggested a role for erythrocytes as potential mediators of inflammatory processes. In contrast to the cloned receptors described, a promiscuous receptor on red blood cells has been characterized, that binds a wide variety of inflammatory peptides of both the CXC and CC groups within the chemokine superfamily.[41,69]

The human erythrocyte chemokine receptor, which was originally postulated to be a 'sink' for IL-8[70] binds the CXC chemokines IL-8, MGSA and PF-4, and the CC chemokines MCP-1 and RANTES with equal high affinity.[24] Other experiments show that the RBC-bound IL-8 (and most likely other chemokines) does not induce signalling in target cells and that chemokines bound to the red cell surface are inaccessible to their normal target inflammatory cells.[69] Thus, the major role for the red cell chemokine receptor may be one of a clearance receptor for chemotactic and inflammatory peptides in the blood. Due to the broad ligand specificity of the red blood cell receptor, it has been designated the multispecific chemokine (CK) receptor.[41]

The fact that the molecular mass of the erythrocyte CK receptor is at least 19 kDa smaller than the molecular mass of the cloned IL-8 receptors, as well as the ability of the CK receptor to bind to a variety of chemokines, supports the idea that this receptor has a different structure compared to the cloned IL-8 receptors. Moreover, the CK receptor showed no sensitivity to GTP or to GTPγS at concentrations which resulted in a 50% reduction in IL-8 binding to plasma membranes prepared from cells transfected with one of the cloned IL-8 receptors.[71] These data do not support the idea that the CK receptor is G-protein linked. It is still possible, however, that the erythrocyte CK receptor retains the seven transmembrane domain characteristic of this family of receptors, but that it is uncoupled from its guanine nucleotide transducing unit. Alternatively, the erythrocyte CK receptor may have a unique three-dimensional protein structure compared with that of the cloned IL-8 receptors. Evidence in support of either of these two possibilities awaits purification and sequencing of this protein.[41]

### Structure–Activity Relationships of Chemokines

The compact, symmetrical nature of the familiar IL-8 dimer structure led to the widespread presumption that the dimer form must be important for function. This notion is extended further by the finding that MIP-1β has an entirely different mode of dimerization. Thus it has been suggested that all the CXC chemokines have a six-stranded β-sheet dimer (three antiparallel β-strands from each monomer), whereas all the CC chemokines an end-on-end dimer structure and, moreover, that this structural difference may account for the functional differences between the two families. Lusti-Narasimhan *et al.*[72] and Clark-Lewis *et al.*[36] present the case from an opposite viewpoint: that the functional form is the monomer and dimerization is not relevant for interaction with the functional receptor. There are several reasons for this hypothesis. First, ligands for the GPCRs are mostly small peptides or nonpeptide hormones and mediators. Therefore, it seems unlikely that the chemokine receptors accommodate chemokine dimers. Second, protein structures are determined at high concentration and thus the thermodynamics will strongly favour dimerization.[36] Third, IL-8, which contains N-methyl-leucine 25, is always monomeric and yet remains active.[72]

### Structure–Activity of CXC Chemokines

### The ELR motif

As already mentioned in the introduction of this section the ELR motif is the most critical region for interaction with the IL-8R.[36] Mutagenesis and peptide synthesis showed that out of all of the charged residues, in IL-8 only the amino-terminal Glu4-Leu5-Arg6 (ELR) sequence was absolutely required. The ELR region can be modified such that the receptor binding is retained but activity is lost. It is striking that these antagonists have much lower receptor affinity than IL-8, because usually antagonists have higher binding affinity than agonists. Therefore, this indicates that the ELR motif is both a binding and receptor-activation motif.[73] Multiple substitutions showed that all three residues of the ELR motif were highly sensitive to modification, with the order of sensitivity being $R \gg E > L$.[36] Additionally, the ELR conformation and side chain integrity is critical, as substitution of NMe-Leu and NMe-Arg, or single D-amino acid substitutions greatly reduced activity. Adding 'spacer' residues, either Glu or Ala, between the ELR and the cysteine at position 7 resulted in loss of activity with only some residual binding. ELR effects are subtly context-dependent since PF-4, but not γIP-10 or MCP-1, binds to IL-8 receptors and activates neutrophils when its N-terminus is modified to contain ELR.[74]

ELR-containing N-terminal peptides of IL-8 lack agonist activity, indicating that ELR may be necessary but not sufficient for receptor activation. The α helical C-terminal domain of IL-8 also contains determinants for receptor activation.[27]

### The loop region

The loop region, consisting of amino acids 10–22, was generally not affected by single substitutions. However, experiments with hybrid proteins of IL-8 and γIP-10 demonstrated that this entire region was critical for IL-8 activity. The residues close to the $NH_2$-terminal end of the loop, i.e. close to cysteine 9, were the most critical.[36] The major difference between the single substitution and hybrid strategies is that the hybrids had multiple replacements. Thus, only when several substitutions were made, significant effects were observed. Taken together, the results suggest that the N-terminal loop comprises a secondary binding site.[27] The amino acids 18–22, however, do not appear to be essential, as multiple substitutions in this region failed to affect activity. Phe21 makes aromatic contacts with Tyr13, Phe17, and Trp57 and may have a structural role. Nevertheless, the possibility that Phe21 has hydrophobic or aromatic contacts with the receptor cannot be ruled out.[36]

### The disulphides

When the cysteines that form each disulphide bridge were substituted in pairs with the cysteine isostere, $\alpha$-aminobutyric acid (side chain $CH_2$–$CH_3$), both analogues were inactive and NMR studies showed significant structural perturbation, probably due to loss of the disulphide. Both disulphides are essential for function, indicated by lack of activity of the two analogues. However, they do not seem to be essential for chemokine function in general, as lymphotactin, which lacks both disulphide bridges, is still a chemoattractant.[27]

### The 30–35 region

His33 was analysed extensively due to its interaction with the CXC region and proximity to the two disulphides and the ELR motif, but various substitutions had no effect on activity. Further analogues showed that the Gly31–Pro32 motif in the 30–35 region was essential. This motif determines the structure of the 30–35 region, and, most likely, also the 7–34 disulphide. The 7–34 disulphide would in turn influence the conformation of the ELR motif.

### Structure–Activity of the CC Chemokines

Based on the sequence homology of chemokines, Clark-Lewis and co-workers[36] hypothesized that there could be similarities in the way that CXC and CC chemokines interact with their receptors. They speculated that the N-terminal region would be critical and that secondary sites would be necessary. However, instead of just three residues as in the CXC chemokines, the entire 10 residues that are $NH_2$-terminal to the first cysteines were important. Deletion of the first residue of MCP-1 markedly decreased activity. This contrasts with the CXC chemokines, where only the ELRCXC motif of the N-terminal region is essential. For MCP-1, addition of a residue to the $NH_2$-terminal or acetylation of the $NH_2$-terminal glutamine resulted in loss of activity. Analogues with the $NH_2$-terminal residue converted to Asn, or residues with nonpolar side chains of varying size, had equivalent activity to native MCP-1. Analogues that had either one, two or three residues deleted from the $NH_2$-terminal had lower binding affinity and activity than full-length native MCP-1. However, MCP-1, 5–76 had surprisingly significant activity and bound to the MCP-1 receptor.[36] Further deletions resulted in analogues that had significant binding to the receptor but no functional activity. Clark-Lewis and co-workers[36] propose the existence of an activation region and a receptor binding region that comprise residues 1–5 and 7–10 respectively. Truncation of the $NH_2$-terminal region (up to the first cysteine) of MCP-1 resulted in MCP-1, 11–76, which had residual binding activity, suggesting that a second region binds, although with low affinity, independently of residues 1–10.[75] This contrasts with the CXC chemokines, where truncation of the ELR motif resulted in absence of receptor binding. Experiments with hybrids of MCP-1 and MCP-3 led to the suggestion that the $NH_2$-terminal is not sufficient to determine activity, and that the $NH_2$-terminal binding site and secondary sites complement each other to give maximal binding and activity. The CC chemokines were analysed for the chemotactic activity on monocytes and THP-1 cells. The order of potency was MCP-1, MCP-3, MCP-2, RANTES, MIP-1$\alpha$ and MIP-1$\beta$. It was found that MCP-3 and MCP-2 both stimulate chemotaxis, enzyme release, and intracellular calcium induction in monocytes and THP-1 cells and enzyme release in monocytes. MCP-3 is always the more potent of the two. MCP-3 and MCP-2 appear to be functionally similar and both stimulate basophils, eosinophils, and lymphocytes, as well as monocytes. This is in contrast to published findings suggesting a distinct mechanism of action for MCP-2.[36]

### Mutation of Leu25 and Val27 in IL-8

Examination of the sequences of the CXC chemokines reveals that the highly conserved leucine, corresponding to Leu25 in IL-8, is in all cases replaced by a tyrosine in CC chemokines. There is also a high degree of conservation among the CXC chemokines of the adjacent Val27 residue, which protrudes from the same side of the $\beta$-sheet as Leu25. In RANTES, Val27 is also replaced by a tyrosine. Mutation of either Leu25 or Val27 to tyrosine residues results in a decrease in affinity for the IL-8 receptor on neutrophils and a simultaneous decrease in the physiological response of neutrophils. The mutation Leu25 > Tyr has the more dramatic effect, showing a 100-fold drop in receptor binding. This mutation in IL-8 induces a novel monocyte chemotaxis activity, indicating that Leu25 and Val27 are important in the interaction not only with the neutrophil IL-8 receptors, but also with the monocyte CC chemokine receptors.[72] Previous studies have already shown that substitution of Tyr28 and Arg30 in the first β-sheet of MCP-1 with the corresponding residues found in IL-8 resulted in a switch from monocyte to neutrophil specificity for the mutated molecule.[76]

### Transendothelial Migration of Leukocytes

Recruitment of leukocytes to sites of localized inflammation is a feature of several human disease states. There is a diverse range of leukocyte types and functions, and the different cells appear to migrate to the appropriate site in an impressively ordered and regulated manner. It is this highly elaborate process of cell influx that is the hallmark of the inflammatory process.[77] The histology of inflamed sites can differ markedly. The acute infiltrate in common bacterial infections, or after local deposition of IgG immune complexes is mainly neutrophil, whereas mononuclear cells predominate in infections by intracellular pathogens, and in delayed-type hypersensitivity. By contrast, eosinophil and basophil leukocytes are prominent in inflammatory reactions that follow immediate-type allergy, certain parasitic infections and autoimmune events.[78] Moreover, increased numbers of eosinophils have been reported in the lung tissues and airways of patients affected by a number of respiratory pathologies including nasal polyposis and asthma.[39]

The *in vivo* requirements for a trafficking cell are quite complicated, and broadly include four distinct components: circulation, adhesion, diapedesis (migration through junctions between endothelial cells), and migration.[39] Until recently, the mechanisms for the recruitment of a given type of leukocyte into inflamed tissue remained largely a mystery, since most inflammatory cytokines, mediators and chemoattractants have little target cell selectivity. It was suggested that some selectivity may result from the type of adhesion receptors expressed on endothelial cells, e.g. vascular cell adhesion molecule (VCAM) recognition of very late antigen 4 (VLA-4), which is present on monocytes, basophils and eosinophils, but not neutrophils. Furthermore, priming by haematopoietic growth factors can also influence the type of cellular infiltrate. For instance, IL-3 and IL-5 markedly enhance the migration and release responses of eosinophil and basophil leukocytes, but do not affect neutrophils.[78]

In the past few years, an improved understanding of cell adhesion and intracellular signalling have helped to unravel some of the details of this important, but complex, process.[77] First, leukocytes must overcome haemodynamic forces in order to adhere to the endothelial cell surface, lining the typical vessel wall. Subsequently, they must 'crawl their way' along the endothelial surface, migrate through junctions between endothelial cells (the process of diapedesis), and penetrate the basement membrane before gaining entry into, and migration through, the tissue spaces.[39] The inflammatory process is now thought to be a multi-step phenomenon with contributions from four different families of adhesion molecules, including the selectins and their related carbohydrate and glycoprotein ligands, the integrins and their related immunoglobulin superfamily ligands, and a diverse set of small signalling molecules known as chemokines and their respective receptors.[77] The coordinated expression of adhesion receptors on the surface of the leukocytes and their counterreceptors on the surface of endothelial cells are thought to be a key link in the process. Models of the adhesion component of leukocyte trafficking have been refined into a 'three step' process comprising: (a) rolling of leukocytes along the vasculature (mediated through transient interactions between so-called selectin proteins and their carbohydrate ligands), followed by (b) activation of the cell (induced by classical chemoattractants or chemokines) resulting in firm adhesion (mediated through integrin molecules) leading ultimately to (c) extravasation (crawling along the endothelium, diapedesis, and migration into tissues), presumably in response to a chemoattractant gradient.[39] A key feature is that selectin–carbohydrate, chemoattractant–receptor, and integrin–immunoglobulin family interactions act in sequence, not in parallel. This concept has been confirmed by the observation that inhibition of any one of these steps, with e.g. selectin antagonists, gives essentially complete rather than partial, inhibition of neutrophil and monocyte migration.[79] An important consequence of a sequence of steps, at any one of which are choices of multiple receptors or ligands that have distinct distributions on leukocyte subpopulations or endothelium, is that it provides great combinatorial diversity for regulating the selectivity of leukocyte localization *in vivo*, as has been emphasized in several reviews.[80–82] Each type of leukocyte responds to a particular set of area code signals. Inflammation alters the expression and location of the signals on vascular endothelium. Chemoattractants provide the greatest number of molecular choices and thus the greatest cellular specificity.[79]

The 'three step' process discussed above is oversimplified and refinements to this model are required. Firstly, selectins actually mediate two steps, initial tethering to the vessel wall and rolling, which can be distinguished for E-selectin (see Selectins) by dependence on different classes of neutrophil ligands. Thus, some selectin–ligand combinations may be important in tethering and others in rolling. Leukocytes in the bloodstream travel about 1 000 microns per second, much too fast for them to sense the chemotactic factor emanating from a site of damage or infection. The selectins and their carbohydrate ligands have been found to mediate the initial decelerating event, which is characterized by the tethering and subsequent rolling that allows the leukocyte to test the microenvironment adjacent to the inflammatory site.[77]

Secondly, the steps are overlapping, rather than strictly sequential. Although L-selectin is shed from neutrophils soon after activation, ligands for E-selectin remains on the neutrophil surface, and thus interactions with E-selectin will probably persist until transendothelial migration is completed.[79]

### Selectins

The selectins or lectin cellular adhesion molecules, include the molecules L-selectin, P-selectin and E-selectin. They are transmembrane molecules, with a number of extracellular domains homologous to those seen in the complement receptors. The extracellular region also has a domain related to the EGF-receptor (epidermal growth factor) and a N-terminal domain which has lectin-like properties (i.e. it binds to carbohydrate residues).[83] L-selectin is expressed on all circulating leukocytes, except for a subpopulation of memory lymphocytes. P-selectin is stored preformed in the Weibel-Palade bodies of endothelial cells and the α granules of platelets. In response to mediators of acute inflammation, such as thrombin or histamine, P-selectin is rapidly mobilized to the plasma membrane to bind neutrophils and monocytes. E-selectin is induced on vascular endothelial cells by cytokines such as IL-1, lipopolysaccharide or TNF and requires *de novo* mRNA and protein synthesis. Selectins mediate a function unique to the vasculature, the attachment or tethering of flowing leukocytes to the vessel wall through labile adhesions that permit leukocytes to roll in the direction of the flow.

### Carbohydrates and mucin-like molecules

Selectins appear to recognize a sialylated carbohydrate determinant on their counterreceptors. The carbohydrate ligands for L- and P-selectin are O-linked to specific mucin-like molecules. Mucins are serine- and threonine-rich proteins that are heavily O-glycosylated and have an extended structure.

### Chemoattractants

Chemoattractants are important in activation of integrin adhesiveness and in directing the migration of leukocytes. In chemotaxis, cells move in the direction of increasing concentration of a chemoattractant, which typically is a soluble molecule that can diffuse away from the site of its production, where its concentration is highest.[84] Leukocytes, which can sense a difference of 1% in chemokine concentration across their diameter, move steadily in the direction of the chemoattractant. As mentioned earlier, the classical leukocyte chemoattractant acts broadly, on neutrophils, eosinophils, basophils, and monocytes, whereas the chemokines have specificity for leukocyte subsets.[79] This suggests that the chemokines may be centrally involved in specific (transendothelial) migration of leukocyte subsets. The CC chemokine RANTES is a chemoattractant for memory T cells *in vitro* and human MIP-1α and MIP-1β have been found to be chemoattractant for distinct subpopulations of lymphocytes including naive T-cells and B-cells. The CC chemokines MCP-1 and C10 are thought to induce T cell migration[39] just as some of the CXC chemokines e.g. IL-8 and IP-10. The C chemokine, lymphotactin, also shows T-lymphocyte chemoattractant activity. Furthermore, some of the CC chemokines are potent promigratory signals for basophils and eosinophils, findings which may be relevant to the understanding of allergy and asthma.[39]

It has long been discussed whether chemoattractants can act in the blood stream, where they would be rapidly diluted and swept downstream by bloodflow. Tethering and rolling of leukocytes through selectins will enhance exposure to chemoattractants by prolonging leukocyte contact with the vessel wall. However, retention of chemoattractants at their site of production by noncovalent interactions with molecules on the vessel wall and within the inflammatory site may also be important. Heparin binding sites on chemokines provide a mechanism for retention in the extracellular matrix, enhancement of concentration gradients, and perhaps presentation of chemokines on the endothelium to circulating leukocytes.[85]

### Chemoattractant receptors

Leukocyte chemoattractant receptors have multiple functions. They do not only direct migration, but also activate integrin adhesiveness and stimulate degranulation, shape change, actin polymerization, and the respiratory burst.[79] As mentioned earlier, chemoattractant receptors are G-protein coupled receptors that span the membrane seven times. Neutrophils and lymphocytes express $G\alpha_{i2}$ and $G\alpha_{i3}$ subunits. The $G\alpha$ subunits of the $\alpha_i$ class are ADP-ribosylated and irreversible inactivated by pertussis toxin. All of the biological effects of leukocyte chemoattractants are inhibited by pertussis toxin. Coupling through $G\alpha_i$ subunits has been confirmed by reconstitution in transfected cells.[79]

### Integrins

Integrins are perhaps the most versatile of the several adhesion molecules. They are integral membrane proteins that help to bind cells to the extracellular matrix. Each member of this large family of molecules consists of two noncovalently bound polypeptides (α and β), both of which traverse the membrane. They fall into three main sub-families, depending on whether they have a β1 chain, a β2 chain or a β3 chain. Recent discoveries suggest that the assortment of a chains with β chains is not quite as precise as originally thought. Broadly speaking the β1-integrins are involved in binding of cells to extracellular matrix, the β2-integrins are involved in leukocyte adhesion to endothelium or to other immune cells, and the β3-integrins are involved in the interactions of platelets and neutrophils at inflammatory sites or sites of vascular damage. Integrin adhesiveness can be rapidly regulated by the cells on which they are expressed. Thus far, the best candidates for activation of integrin adhesiveness within the vasculature are chemoattractants. It is likely that the increased adhesiveness of integrins, such as Mac-1 and lymphocyte function-associated antigen 1 (LFA-1), is due to a conformational change in the integrins upon activation.

### Immunoglobulin superfamily members on endothelium as integrin ligands

Several different immunoglobulin superfamily (IgSF) members, expressed on endothelium bind to integrins expressed on leukocytes. ICAM-1 (intercellular adhesion molecule 1), ICAM-2, and ICAM-3 are products of distinct and homologous genes and were all initially identified by their ability to interact with LFA-1. Induction of ICAM-1 on endothelium and other cells by inflammatory cytokines may increase cell–cell interactions and leukocyte extravasation at inflammatory sites, whereas constitutive expression of ICAM-2 may be important for leukocyte trafficking in uninflamed tissues, as in lymphocyte recirculation. Vascular cell adhesion molecule 1 (VCAM-1) is inducible by cytokines on endothelial cells and on a more restricted subset of nonvascular cells than ICAM-1.[79]

### The role of chemokines in chemotaxis

The selective chemoattractant activities of the chemokines make them ideal candidates to play a key role in the 'sorting' problem of leukocyte trafficking, i.e. getting the correct subpopulation of cells to migrate into the tissues. The chemokines may be even more ideally adapted to directing leukocyte trafficking, because some of these proteins can also promote cell subtype specific adhesion.[39] Taub and colleagues, have reported that both MIP-1α and -1β, as well as RANTES and γIP-10, increase the adhesive properties of the cells for which they are chemoattractant.[39] However, if chemokines play an important part in attracting rolling leukocytes to the inflammatory site, then it is likely that they would form a chemotactic gradient; however, until a few years ago it was unclear how they could form an appropriate gradient under the conditions of vascular flow. An initial clue to the way in which a chemokine gradient could be formed came from examination of the chemokine sequences. All of the chemokines have positively charged domains capable of binding the highly negatively charged carbohydrates of proteoglycans, and a number of different chemokines have been shown to be capable of binding immobilized carbohydrates, such as heparin sulphate.[77] Tanaka *et al.*[85] have provided strong evidence for an association between MIP-1β and glycosaminoglycans (GAGs) on the proteoglycan CD44 (Fig. 5). They have shown that MIP-1β is present on lymph node endothelium and that immobilized MIP-1β

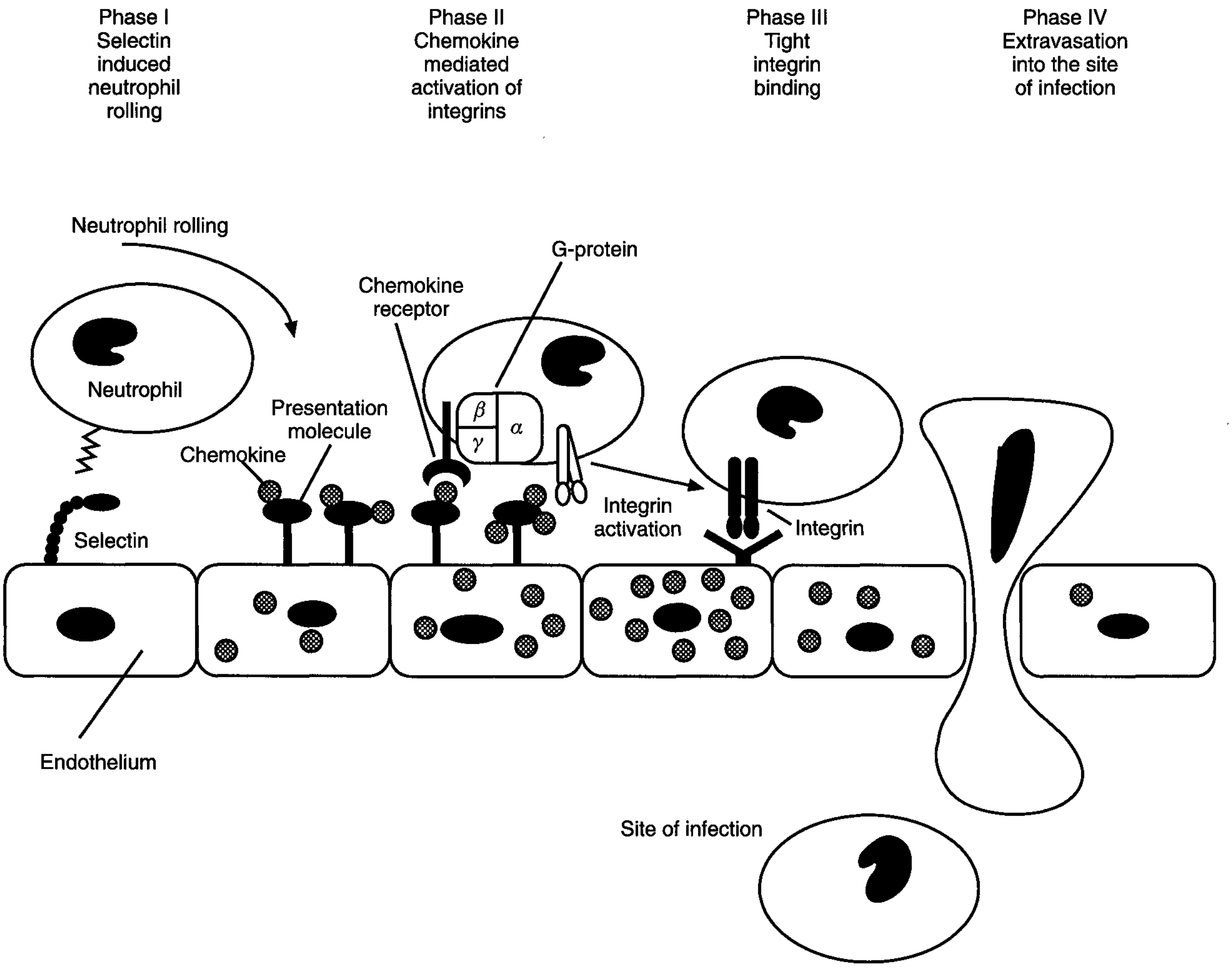

FIG. 5. A hypothetical, multistep model of the extravasation of specific leukocyte subsets near a site of infection. The sequential steps provide the traffic signals that regulate leukocyte localization in the vasculature. Lymphokines produced in response to the pathogen induce changes in the epithelium, including the formation of a gradient of specific chemokines: the gradient may be generated by local production of the chemokines by endothelial cells and their electrostatic attachment to glycosaminoglycan carbohydrates on proteoglycans as CD44.[77,79]

induces binding of T cells to VCAM-1 *in vitro*. In these experiments, MIP-1β was immobilized by binding to proteoglycan: a conjugate of heparin with bovine serum albumin (BSA) and cellular proteoglycan CD44 were both effective. Tanaka *et al.* propose that MIP-1β and other cytokines with glycosaminoglycan-binding sites will bind to and be presented by endothelial proteoglycans to trigger adhesion selectivity not only of lymphocyte subsets, but also for other cell types.[85]

Evidence has now accumulated that chemokines may generally form chemical gradients in an immobilized phase via electrostatic interactions with negatively charged proteoglycans.[39] As a result, it might be convenient to think of chemokines as requiring a scaffolding or presentation molecule in order to interact properly with their related receptor (Fig. 5). This would be an appropriate strategy *in vivo*, as unless the chemotactic gradient was preserved in a solid phase, normal conditions of blood flow would wash away any chemoattractant, and a constant replenishment would be required at the source. As the chemoattractant can now be considered as being sequestered and maintained by stable components of the extracellular matrix, a single release of chemokine (as might occur during platelet degranulation) might be sufficient to initiate the inflammatory cascade. The inflammatory response could then be 'fine tuned' as each cell which traffics through a vessel could leave its own signals bound in solid phase.

The model of chemokine involvement in leukocyte trafficking can now be summarized as follows:

(a) a chemokine, sequestered in solid phase on the endothelial cell surface, is presented as a signal to trap a specific type of leukocyte as the cell is undergoing selectin-mediated rolling along the endothelium;
(b) the leukocyte is selectively activated by the chemokine so that the cell stops rolling and becomes firmly adhered;
(c) the adhered leukocyte 'crawls' along the chemotactic gradient formed by the chemokines on the endothelium;
(d) the leukocyte undergoes diapedesis and migrates into the tissue space, while still responding to a chemotactic gradient.

In the following sections we will discuss certain specific chemokines in relation to asthma and inflammation.

### The Effect of RANTES on the Eosinophil Transendothelial Migration (TEM)

Studies using an *in vitro* model of TEM utilizing eosinophils,[86] showed that eosinophil TEM is in certain ways similar to that of neutrophils. For example, activation of endothelial cells with IL-1 or TNF can significantly increase eosinophil TEM. In contrast, a number of differences between eosinophil TEM and that of neutrophils or other leukocytes have also been observed. Notable among these is the observation that eosinophil active cytokines, including IL-3, IL-5, and GM-CSF, can profoundly potentiate the TEM of eosinophils, while having no effect on neutrophils. These cytokines are not acting as chemoattractants as they need not be present during the TEM assay. Analysis of a host of chemokine molecules has revealed that especially RANTES is an effective eosinophil chemoattractant which has no migration-stimulating properties for neutrophils. The chemokines investigated were IL-8, PF-4, B-TG, γIP-10, MCAF, MIP-1α, RANTES, MIP-1β and I-309. In addition, injection of human RANTES into dog skin has been shown to induce a profound eosinophilic infiltrate.[32] The effect of RANTES was concentration-dependent, was inhibited by antibodies against the CD18 adhesion complex on eosinophils, and was greatly potentiated by exposure of the eosinophils to the priming cytokine, IL-5. Interestingly, the chemokine RANTES did not cause changes in eosinophil adhesion molecule expression, nor did it induce any apparent increase in adhesion of eosinophils to either resting endothelial cells or cultured endothelial cells activated with IL-1.[86] Previous studies showed that CD18 and its endothelial counterligand, ICAM-1, are quite important in TEM of eosinophils across IL-1-activated endothelial cells. Similar conclusions have been derived from studies of lymphocyte and neutrophil TEM. One important difference between eosinophils and neutrophils is that when the CD18 molecule is dysfunctional or absent (as e.g. is the case in leukocyte adhesion deficiency disease) neutrophils do not migrate outside of the vasculature into skin and most other tissues; in these patients, eosinophils are still capable of migrating. Ebisawa *et al.*[86] speculate that the VLA-4/VCAM-1 system may operate as a failsafe mechanism for TEM in cases in which the CD18/ICAM-1 pathway is not functional. It is also possible that the VLA-4/VCAM-1 pathway, which is operative in monocytes and lymphocytes as well, may be utilized during stimulation of the CC chemokine receptor when leukocytes are migrating across activated endothelium *in vivo*.

Asthmatic individuals have elevated levels of eosinophil-priming cytokines in their circulation as well as in the airways; allergen challenge causes dramatic increases in levels of these cytokines. Furthermore, eosinophils of asthmatic subjects display evidence of having been subject to priming *in vivo*. Although the effect of RANTES on BAL eosinophils has not been assessed directly, one may speculate that the synergy between priming cytokine and RANTES chemotaxis would be expected in these BAL eosinophils. It has recently been shown that higher levels of RANTES are found in the BAL fluid of asthmatic individuals than in normal individuals. Dahinden *et al.*[87] reported that MCP-3 is also chemotactic for eosinophils. Although it is approximately one order of magnitude less potent than RANTES in activating eosinophils, MCP-3 must also be considered as having potential relevance to eosinophilic responses *in vivo*.[87]

### The Effect of MCP-3 on Eosinophils and Basophils

In basophils, MCP-3, MCP-1, RANTES and MIP-1α all induced cytosolic free-calcium concentration changes and, with different efficacies, chemotaxis (RANTES = MCP-3 ≫ MCP-1 > MIP-1α), histamine release (MCP-1 = MCP-3 ≫ RANTES > MIP-1α), and $LTC_4$ formation after IL-3 pretreatment (MCP-1 = MCP-3 ≫ RANTES > MIP-1α).[87] Thus, MCP-3 is as effective as MCP-1 as an inducer of mediator release, and as effective as RANTES as a stimulus of basophil migration. In contrast to MCP-1, MCP-3 is also a stimulus for eosinophils, and induces $[Ca^{2+}]_i$ changes and chemotaxis as effectively as RANTES.

MCP-3 has been reported to interact with several CC chemokine receptors, which can be simultaneously or selectively expressed on leukocyte subpopulations.[88] Studies based on desensitization of the calcium flux predicted at least three types of receptors: (1) MCP-1 receptor on monocytes and basophils, (2) selective RANTES receptor on basophils and eosinophils, and (3) selective MIP-1α receptor on basophils, eosinophils, and neutrophils. Results obtained from binding studies using [$^{125}$I]MCP-1 and [$^{125}$I]MIP-1α on monocytes suggested that MCP-3 may also interact with CC CKR1, the MIP-1α/RANTES receptor. Ben-Baruch *et al.*[88] demonstrated that CC CKR1 exhibited even higher binding affinity for [$^{125}$I]MCP-3 than for [$^{125}$I]RANTES and [$^{125}$I]MIP-1α. Thus, MCP-3 may, because of its powerful stimulus of chemotaxis for both eosinophils and basophils, and of histamine and $LTC_4$ release from basophils, play an important role in asthma. MCP-1 might be involved as well, as it is a chemoattractant for, and stimulates histamine and $LTC_4$ release from, basophils very effectively. Moreover, MCP-1 is also found in the bronchial epithelium of asthmatic patients.[89] To date the role of MCP-2 has not been elucidated, but as it is not very potent in chemotaxis and activation, it is thought not to play a critical role in diseases such as asthma.

### Eotaxin

Asthma is often characterized by tissue recruitment of predominantly eosinophils; chemokines acting on eosinophils include certain CC chemokines, e.g. MCP-2, MCP-3, RANTES and MIP-1α. The CXC chemokine IL-8 is also chemoattractive for cytokine-primed eosinophils. However, none of these chemoattractive molecules are eosinophil-specific and their relative importance in selected diseases and experimental animal models for allergy remains unclear.[90] In contrast to the factors discussed so far, eotaxin, a recently described CC chemokine, has been proposed as an eosinophil chemoattractant in a guinea-pig model of allergic airway inflammation.[66,91] Eotaxin appears to be unique among the chemokines since it causes the selective infiltration of eosinophils only, when injected into the skin and when directly administered to the lungs of naive guinea-pigs. In experiments described by Rothenberg *et al.*[90] migrating cells (induced by eotaxin) were > 95% eosinophils.

In 1993, Griffiths-Johnson and colleagues[91] reported the purification of a novel chemokine, 'eotaxin', from bronchoalveolar lavage (BAL) fluid collected 3 h after aerosol allergen challenge of actively sensitized guinea-pigs. The HPLC fraction that showed eosinophil chemoattractant activity, showed no permeability-increasing activity. *In vitro*, eotaxin induced increases in $[Ca^{2+}]_i$ and induced a dose-related eosinophil aggregation. *In vivo*, eotaxin induced substantial eosinophil accumulation when injected in the skin of naive guinea-pigs. No significant changes in the number of neutrophils or mononuclear cells were observed.[91]

Eotaxin consists of 73 amino acids and is a member of the CC branch of chemokines. Surprisingly, the greatest homology is with human MCP-1 (53%), MCP-2 (54%) and MCP-3 (51%) with respect to the amino acid sequence. As mentioned earlier MCP-1 has been reported to be inactive on human eosinophils. Homology with other human CC chemokines is rather low: MIP-1β (37%), MIP-1α (31%), and RANTES (26%). The latter two proteins have been shown to be potent eosinophil activators *in vitro*, whereas MIP-1β activates lymphocytes *in vitro*, but apparently not eosinophils.[66] Due to the high homology with MCP-3 and the fact that MCP-3 and eotaxin are both causing eosinophil chemotaxis, it was first thought that guinea-pig eotaxin is the homologue of human MCP-3. This, however, seems unlikely since eotaxin does not share the chemotactic activity with MCP-3 on other cells than eosinophils.

Rothenberg *et al.*[90] have identified a murine eotaxin, and the structural similarities between murine (mouse) and guinea-pig eotaxin indicate that both are more closely related to each other than to other members of the CC family of chemokines. For example, each protein contains several unique features including a gap in the alignment with the MCPs of two amino acids near the N-terminal end of the protein and the conservation of basic amino acids near the C-terminal end that distinguish it from other CC chemokines. It is also noteworthy that the N-terminal end of MCP-1, including the N-terminal Gln, which has been shown to be critical for monocyte activity, is replaced by a His in both murine and guinea-pig eotaxin. These comparisons suggest that eotaxin is a distinct cytokine and not a homologue of a known member of the family.

### Eotaxin mRNA expression in different organs

As would be expected, eotaxin mRNA is constitutively expressed in mucosal tissues that normally contain eosinophils (skin, lung, and intestinal tract).[90] Nonetheless, expression of murine eotaxin is also seen in thymus, lymph node, and muscle where resident eosinophils are rare. This pattern of mRNA tissue distribution is similar to that seen in guinea-pigs, although mice have higher expression in the thymus and skin and guinea-pigs have higher expression in the lung.[92] Northern blot analyses of total RNA isolated from different guinea-pig tissue samples revealed easily detectable constitutive expression of eotaxin in the lung. Lower levels were detectable in the intestines, stomach, heart, thymus, spleen, liver, testes, and kidney. In addition, no RNA was detectable in the brain, bone marrow, or skin.[92] The finding of constitutive eotaxin mRNA in mucosal tissues where eosinophils are predominantly located (lung and intestines), suggests that eotaxin may play a role in the normal tissue homing and turnover of eosinophils.

The unexpected expression of eotaxin mRNA in lymphoid tissue and muscle suggests that eotaxin may effect other cell types, because eosinophils are normally not present in these tissues, and that eotaxin might therefore have a more widespread function. The expression in the thymus and lymph node suggests that eotaxin may direct lymphocyte homing.[90] Although the eotaxin gene is expressed at relatively high levels in the lungs of healthy guinea-pigs without airway inflammation, the chemotactic activity ascribed to eotaxin has been reported to be undetectable in the bronchoalveolar fluid of non-antigen-challenged guinea-pigs. Thus, eotaxin mRNA is constitutively expressed at easily detectable levels in the lung, when eotaxin activity is still undetectable. After antigen challenge, eotaxin gene expression in the lung is further increased during the early part of the late phase response. Thus, up-regulation of gene expression, and not constitutive expression, is associated with the pathogenesis of airway disease.[92] The up-regulation of eotaxin mRNA as well as protein after allergen challenge shows that the response is, at least to a large extent, at the level of transcription rather than translation of the existing mRNA, although the factor responsible for this up-regulation is unknown.

Eotaxin is likely to act in parallel with other cytokines generated during the late phase response. For example, IL-5 can prime eosinophils to respond to another CC chemokine, RANTES, and can promote eosinophil tissue survival and activation.[92] The CC chemokines have also been implicated in wound healing which may be important in the subepithelial basement membrane fibrosis that is a prominent feature of the asthmatic lung.

Eosinophils contain an armory of chemicals necessary for killing parasites. These chemicals have been implicated in the damage to airway epithelium that occurs in asthma and may relate to the observed changes in airway function. Rothenberg *et al.*[92] suggest that eotaxin should be considered as a potentially important endogenous mediator of eosinophil accumulation *in vivo*. In particular, eotaxin and related molecules may be involved in both eosinophil accumulation and in chronic structural changes in the asthmatic lung.

Subsequent to the discovery of guinea pig and murine eotaxin, a research team at LeukoSite (Cambridge, MA) very recently identified human eotaxin, examined its chemotactic activity and characterized its binding to an eosinophil receptor, distinct from the CC chemokine receptors CC CKR1 (MIP-1α/RANTES receptor) and CC CKR2A,B (MCP-1 receptor). Human eotaxin manifested a powerful and selective chemotactic activity towards eosinophils in both *in vitro* and *in vivo* assays. The fact that the chemokines are a 'hot topic' is shown by the unusual situation that human eotaxin was already available on the market[93] before its identification and functional characteristics had been published. It was only at the beginning of 1996 that the cloning and functional characterization of human eotaxin was reported by Ponath *et al.*[94]

### CC Chemokines as a Target for New Drug Therapy in Asthma

To date, no studies concerning strategies antagonizing chemokines for asthma therapy are available. The investigations on chemokines so far, have mainly focused on the discovery of new chemokines and their receptors, and the understanding of their function. It has been reported that glucocorticoids inhibited the epithelial expression of RANTES.[95]

Glucocorticoids have been used in therapy for many years and they are currently the first choice treatment for asthmatic patients. These steroids however, have many functions e.g. inhibition of the production and activity of many cytokines, reduced generation of eicosanoids and PAF, reduced cyclooxygenase-2 expression, increased $\beta_2$ expression, reduced vasodilatation and decreased fluid exudation. As a result of this wide variety of functions, corticosteroids can cause severe side effects e.g. osteoporosis, suppression of endogenous glucocorticoid synthesis, poor wound healing, superinfections, tendency to hyperglycaemia and thinning of the skin.[96] These undesired effects can be reduced by local application. In severe asthma however, the steroids are administered systemically.

New therapies are the development of drugs that could aim at a selective inhibition of the migration of leukocytes involved in a specific disease. As discussed in this review, chemokines are thought to play a major role in the recruitment of these leukocytes and therefore, drugs that modify the production and/or function of these chemokines might be worth investigating. In asthma the attention should be focused on the chemokines that predominantly cause the recruitment of eosinophils. Modifications are possible at several levels. Firstly, specific antibodies can be developed. For IL-8 there is already an antibody available which selectively blocks the IL-8 function. Antibodies for eotaxin and maybe also for RANTES and MCP-3 may be successful. However, the use of antibodies might in practice not be effective, due to typical pharmaceutical constraints. Secondly, the development of antagonists for the receptors involved, should be considered. There are probably several different chemokines involved, all contributing to some degree. Thus, antagonizing the promiscuous receptors may therefore be the most effective. Preliminary results were obtained by Wells *et al.*[68] They identified a series of variants of the CC chemokine RANTES that are potent receptor antagonists. These molecules are active in the low nanomolar range, and are able to block CC chemokine effects on purified human cells *in vitro*. Whether these antagonists will also be able to block CC chemokines effects *in vivo* remains to be elucidated. Thirdly, the production of chemokines can be inhibited by the use of antisense RNA. In this way translation of the mRNA is prevented and thus the production of the target chemokine. Depending on the homology between the nucleotide sequences of different chemokines, this method might be very selective. From these three options, the development of chemokine antagonists seems the most promising, as they have already been shown to be effective *in vitro*. Furthermore, their use in practice is not limited due to typical constraints, as is the case for antibodies and peptidic compounds.

### References

1. Voss H-P. Long-acting $\beta_2$-adrenoceptor agonists in asthma: molecular pharmacological aspects. Thesis, Vrije Universiteit Amsterdam, 1994.
2. Barnes PJ. Anti-inflammatory therapy. *Annu Rev Med* 1993; **44**: 229–242.
3. Virchow JC Jr, Kroegel C, Walker C, Matthys H. Cellular and immunological markers of allergic and intrinsic bronchial asthma. *Lung* 1994; **172**: 313–334.
4. Shelhamer JH, Levine SJ, Wu T, Jacoby DB, Kaliner MA, Rennard SI. NIH conference. Airway inflammation. *Ann Intern Med* 1995; **123**: 288–304.
5. Rang HP, Dale MM, Ritter JM, eds. The respiratory system. In: *Pharmacology*. New York: Churchill Livingstone, 1995; 351–366.
6. Linssen M. Animal models for the selection of anti-inflammatory compounds for the treatment of bronchial hyperreactivity in asthma. Thesis, Vrije Universiteit Amsterdam, 1990.
7. Kay AB, Corrigan CJ. Eosinophils and neutrophils. *Br Med Bull* 1992; **48**: 51–64.
8. Barnes PJ, ed. *Asthma*. Vol 48. London: Churchill Livingstone, 1992.
9. Doelman CJA. Reactive oxygen species and airway hyperreactivity. Thesis, Vrije Universiteit Amsterdam, 1991.
10. Kirby JG, Hargreave FE, Gleich CJ, O'Byrne PM. Bronchoalveolar cell profiles of asthmatic and nonasthmatic subjects. *Am Rev Respir Dis* 1987; **136**: 379–383.
11. Wardlaw AJ, Dunnete S, Gleich GJ, Collins JV, Kay AB. Eosinophils and mast cells in bronchoalveolar lavage in subjects with mild asthma. *Am Rev Respir Dis* 1988; **137**: 62–69.
12. Ädelroth E, Rosenhall L, Johansson SÅ, Linden M, Venge P. Inflammatory cells and eosinophilic activity in asthmatics investigated by bronchoalveolar lavage: the effects of anti-asthmatic treatment with budesonide or terbutaline. *Am Rev Respir Dis* 1990; **142**: 91–99.
13. Jeffrey PK, Wardlaw AJ, Nelson FC, Collins JV, Kay AB. Bronchial biopsies in asthma. An ultrastructural, quantitative study and correlation with hyperreactivity. *Am Rev Respir Dis* 1989; **140**: 1745–1753.
14. Beasley R, Roche WR, Roberts JA, Holgate ST. Cellular events in the bronchi in mild asthma and after bronchial provocation. *Am Rev Respir Dis* 1989; **139**: 806–817.
15. O'Byrne PM. What is asthma? An update on the mechanisms. *J Invest Allergol Clin Immunol* 1995; **5**: 6–11.
16. Azzawi M, Bradley B, Jeffery PK, Frew AJ, Wardlaw AJ, Knowles G, Assoufi B, Collins JV, Durham S, Kay AB. Identification of activated T-lymphocytes and eosinophils in bronchial biopsies in stable atopic asthma. *Am Rev Respir Dis* 1990; **142**: 1407–1413.
17. Bochner BS, Undem BJ, Lichtenstein LM. Immunological aspects of allergic asthma. *Annu Rev Immunol* 1994; **12**: 295–335.
18. Dale HH, Laidlaw PP. The physiological action of β-imidazolylethylamine. *J Physiol (London)* 1910; **41**: 318–344.
19. Schwartz L, Huff T. Biology of mast cells and basophils. In: Middleton E, Reed CE, Ellis EF, Adkinson Jr NF, Yunginger JW, Busse W, eds. *Allergy Principles and Practice*. St Louis: Mosby, 1993; 135–168.
20. Henocq E, Vargaftig BB. Accumulation of eosinophils in response to intracutaneous PAF-acether and allergen in man. *Lancet* 1986; **2**: 1378–1379.
21. Gundel RH, Letts LG, Gleich GJ. Human eosinophil major basic protein induces airway constriction and airway hyperresponsiveness in primates. *J Clin Invest* 1991; **87**: 1470–1473.
22. Baggiolini M, Dewald B, Moser B. Interleukin-8 and related chemotactic cytokines: CXC and CC chemokines. *Adv Immunol* 1994; **55**: 97–179.
23. Strieter RM, Koch AE, Antony VB, Fick Jr RB, Stradiford TJ, Kunkel SL. The immunopathology of chemotactic cytokines: the role of interleukin-8 and monocyte chemoattractant protein-1. *J Lab Clin Med* 1994; **123**: 183–197.
24. Horuk R. Molecular properties of the chemokine receptor family. *TIPS* 1994; **15**: 159–165.
25. Pruzanski W, Vadas P, eds. *Novel Molecular Approaches to Anti-inflammatory Theory*. Vol. 46. Basel: Birkhäuser Verlag, 1995; 1–22.
26. Boyden Jr SE. The chemotactic effects of mixtures of antibody and antigen on polymorphonuclear leukocytes. *J Exp Med* 1962; **115**: 453–466.
27. Murphy PM. The molecular biology of leukocyte chemoattractant receptors. *Annu Rev Immunol* 1994; **12**: 593–633.
28. Oppenheim JJ, Zachariae COC, Mukaida N, Matsushima K. Properties of the novel proinflammatory supergene 'intercrine' cytokine family. *Annu Rev Immunol* 1991; **9**: 617–648.
29. Luster AS, Unkeless JC, Ravetch JV. Gamma-interferon transcriptionally regulates an early-response gene containing homology to platelet proteins. *Nature* 1985; **315**: 672–676.
30. Yoshimura TK, Matsushima K, Tanaka S, Robinson EA, Appella E, Oppenheim JJ, Leonard EJ. Purification of a human monocyte-derived neutrophil chemotactic factor that shares sequence homology with other host defense cytokines. *Proc Natl Acad Sci USA* 1987; **84**: 9233–9237.
31. Yoshimura TK, Matsushima K, Oppenheim JJ, Leonard EJ. Neutrophil chemotactic factor produced by lipopolysaccharide (LPS)-stimulated human blood mononuclear leukocytes: partial characterization and separation from interleukin 1 (IL 1). *J Immunol* 1987; **139**: 788–793.
32. Meurer R, Riper GV, Feeney W, Cunningham P, Hora Jr D, Springer MS, MacIntyre DE, Rosen H. Formation of eosinophilic and monocytic intradermal inflammatory sites in the dog by injection of human RANTES but not human monocyte chemoattractant protein 1, human macrophage inflammatory protein 1α, of human interleukin 8. *J Exp Med* 1993; **178**: 1913–1921.

33. Kelner GS, Kennedy J, Bacon KB, Kleyensteuber S, Largaespada DA, Jenkins NA, Copeland NG, Bazan JF, Moore KW, Schall TJ, Zlotnik A. Lymphotactin: a cytokine that represents a new class of chemokine. *Science* 1994; **266**: 1395–1399.
34. Kelner G, Zlotnik A. Cytokine production profile of early thymocytes and the characterization of a new class of chemokine. *J Leuk Biol* 1995; **57**: 778–781.
35. Kennedy J, Kelner GS, Kleyensteuber S, Schall TJ, Weiss MC, Yssel H, Schneider PV, Cocks BG, Bacon KB, Zlotnik A. Molecular cloning and functional characterization of human lymphotactin. *J Immunol* 1995; **155**: 203–209.
36. Clark-Lewis I, Kim K-S, Rajarathnam K, Gong J-H, Dewald B, Moser B, Baggiolini M, Sykes BD. Structure-activity relationships of chemokines. *J Leuk Biol* 1995; **57**: 703–711.
37. Farber JM. HuMIG: a new member of the chemokine family of cytokines. *Biochem Biophys Res Comm* 1993; **192**: 223–230.
38. Proost P, De Wolf-Peeters C, Conings R, Opdenakker G, Billiau A, Van Damme J. Identification of a novel granulocyte chemotactic protein (GCP-1) from human tumor cells: in vitro and in vivo comparison with natural forms of GROa, IP-10, and IL-8. *J Immunol* 1993; **150**: 1000–1010.
39. Schall TJ, Bacon KB. Chemokines, leukocyte trafficking, and inflammation. *Curr Opin Immunol* 1994; **6**: 865–873.
40. Chaudhuri A, Polyakova J, Zbrzezna V, Williams K, Gulati S, Pogo AO. Cloning of glycoprotein D cDNA which encodes the major subunit of the Duffy blood group system and the receptor for the plasmodium vivax malaria parasite. *Proc Natl Acad Sci USA* 1993; **90**: 10793–10797.
41. Horuk R, Colby TJ, Darbonne WC, Schall TJ, Neote K. The human erythrocyte inflammatory peptide (chemokine) receptor. Biochemical characterization, solubilization, and development of a binding assay for the soluble receptor. *Biochemistry* 1993; **32**: 5733–5738.
42. Neote K, DiGregorio D, Mak JY, Horuk R, Schall T. Molecular cloning, functional expression, and signaling characteristics of a C-C chemokine receptor. *Cell* 1993; **72**: 415–425.
43. Schall TJ, Stein B, Gorgone G, Bacon KB. Cytomegalovirus encodes a functional receptor for C-C chemokines. In: McFadden G, ed. *Viroceptors, Virokines and Related Immune Modulators Encoded by DNA Viruses*. RG Landes; Chapter 12.
44. Ahuja SK, Murphy PM. Molecular piracy of mammalian interleukin-8 receptor type B by herpesvirus saimiri. *J Biol Chem* 1993; **268**: 20691–20694.
45. Moser B, Schumacher C, von Tscharner V, Clark-Lewis I, Baggiolini M. Neutrophil-activating peptide 2 and *gro*/melanoma growth-stimulatory activity interact with neutrophil-activating peptide 1/interleukin-8 receptors on human neutrophils. *J Biol Chem* 1991; **266**: 10666–10671.
46. Schumacher C, Clark-Lewis I, Baggiolini M, Moser B. High- and low-affinity binding of GROα and neutrophil-activating peptide 2 to interleukin-8 receptors on human neutrophils. *Proc Natl Acad Sci USA* 1992; **89**: 10542–10546.
47. Baggiolini M, Loetscher P, Moser B. Interleukin-8 and the chemokine family. *Int J Immunopharm* 1995; **17**: 103–108.
48. Kelvin DJ, Michiel DF, Johnston JA, Lloyd AR, Sprenger H, Oppenheim JJ, Wang J-M. Chemokines and serpentines: the molecular biology of chemokine receptors. *J Leuk Biol* 1993; **54**: 604–612.
49. Samanta AK, Oppenheim JJ, Matsushima T. Interleukin 8 (monocyte-derived neutrophil chemotactic factor) dynamically regulates its own receptor expression on human neutrophils. *J Biol Chem* 1990; **265**: 183.
50. Besemer J, Hujber A, Kuhn B. Specific binding, internalization, and degradation of human neutrophil activating factor by human polymorphonuclear leukocytes. *J Biol Chem* 1989; **264**: 17409.
51. Grob PM, David E, Warren TC, DeLeon RP, Farina PR, Homon CA. Characterization of a receptor for human monocyte-derived neutrophil chemotactic factor/interleukin-8. *J Biol Chem* 1990; **265**: 8311.
52. Chuntharapai A, Kim KJ. Regulation of the expression of IL-8 receptor A/B by IL-8: possible functions of each receptor. *J Immunol* 1995; **155**: 2587–2594.
53. Chuntharapai A, Lee J, Hebert C, Kim KJ. Monoclonal antibodies detect different distribution patterns of IL-8 receptor A and IL-8 receptor B on human peripheral blood leukocytes. *J Immunol* 1994; **153**: 5682.
54. Lee J, Horuk R, Rice GC, Bennett GL, Camerato T, Wood WI. Characterization of two high affinity human interleukin-8 receptors. *J Biol Chem* 1992; **267**: 16283.
55. Loetscher P, Seitz M, Clark-Lewis I, Baggiolini M, Moser B. Both interleukin-8 receptors independently mediate chemotaxis. Jurkat cells transfected with IL-8R1 of IL-8R2 migrate in response to IL-8, GROα and NAP-2. *FEBS Lett* 1994; **341**: 187.
56. Hébert CA, Chuntharapai A, Smith M, Colby T, Kim J, Horuk R. Partial functional mapping of the human interleukin-8 type A receptor. *J Biol Chem* 1993; **268**: 18549–18553.
57. Clark-Lewis I, Schumacher C, Baggiolini M, Moser B. Structure-activity relationships of interleukin-8 determined using chemically synthesized analogs. Critical role for NH2 terminal residues and evidence for uncoupling of neutrophil chemotaxis, exocytosis, and receptor binding activities. *J Biol Chem* 1991; **266**: 23128–23134.
58. Hébert CA, Vitangcol RV, Baker JB. Scanning mutagenesis of interleukin-8 identifies a cluster of residues required for receptor binding. *J Biol Chem* 1991; **266**: 18989–18994.
59. Probst WC, Snyder LA, Schuster DI, Brosius J, Sealfon SC. Sequence alignment of the G-protein coupled receptor family. *DNA Cell Biol* 1992; **11**: 1–20.
60. Gao J-L, Kuhns DB, Tiffany HL, McDermott D, Li X, Francke U, Murphy PM. Structure and functional expression of the human macrophage inflammatory protein 1α/RANTES receptor. *J Exp Med* 1993; **177**: 1421–1427.
61. Wang J-M, Sherry B, Fivash MJ, Kelvin DJ, Oppenheim JJ. Human recombinant macrophage inflammatory protein-1α and -β and monocyte chemotactic and activating factor utilize common and unique receptors on human monocytes. *J Immunol* 1993; **150**: 1–8.
62. Wang J-M, McVicar DW, Oppenheim JJ, Kelvin DJ. Identification of RANTES receptor on human monocytic cells: competition for binding and desensitization by homologous chemotactic cytokines. *J Exp Med* 1993; **177**: 699–705.
63. Charo IF, Myers SJ, Herman A, Franci C, Connolly AJ, Coughlin SR. Molecular cloning and functional expression of two monocyte chemoattractant protein 1 receptors reveals alternative splicing of the carboxyl-terminal tails. *Proc Natl Acad Sci USA* 1994; **91**: 2752–2756.
64. Combadiere C, Ahuja SK, Murphy PM. Cloning and functional expression of a human eosinophil CC chemokine receptor. *J Biol Chem* 1995; **270**: 16491–16494.
65. Combadiere C, Ahuja SK, Murphy PM. Cloning, chromosomal localization, and RNA expression of a human beta chemokine receptor-like gene. *DNA Cell Biol* 1995; **14**: 673–680.
66. Jose PJ, Griffiths-Johnson DA, Collins PD, Walsh DT, Moqbel R, Totty NF, Truong O, Hsuan JJ, Williams TJ. Eotaxin: a potent eosinophil chemoattractant cytokine detected in a guinea pig model for allergic airways inflammation. *J Exp Med* 1994; **179**: 881–887.
67. Raport CJ, Schweickart VL, Chantry D, Eddy Jr RL, Shows TB, Godiska R, Gray PW. New members of the chemokines receptor gene family. *J Leuk Biol* 1996; **59**: 18–23.
68. Wells TNC, Power CA, Lusti-Narasimhan M, Hoogewerf AJ, Cooke RM, Chung C, Peitsch MC, Proudfoot AEI. Selectivity and antagonism of chemokine receptors. *J Leuk Biol* 1996; **59**: 53–60.
69. Neote K, Darbonne W, Ogez J, Horuk R, Schall TJ. Identification of a promiscuous inflammatory peptide receptor on the surface of red blood cells. *J Biol Chem* 1993; **268**: 12247–12249.
70. Darbonne WC, Rice GC, Mohler MA, Apple T, Hébert CA, Valente AJ, Baker JB. Red blood cells are a sink for interleukin 8, a leukocyte chemotaxin. *J Clin Invest* 1991; **88**: 1362–1369.
71. Holmes WE, Lee J, Kuang W-J, Rice G, Wood WI. Structure and functional expression of a human interleukin-8 receptor. *Science* 1991; **253**: 1278–1280.
72. Lusti-Narasimhan M, Power CA, Allet B, Alouani S, Bacon KB, Mermod J-J, Proudfoot AEI, Wells TNC. Mutation of $Leu^{25}$ and $Val^{27}$ introduces CC chemokine activity into interleukin-8. *J Biol Chem* 1995; **270**: 2716–2721.
73. Moser B, Dewald B, Barella L, Schumacher C, Baggiolini M. Interleukin-8 antagonists generated by N-terminal modification. *J Biol Chem* 1993; **268**: 7125.
74. Clark-Lewis I, Dewald B, Greiser T, Moser B, Baggiolini M. Platelet factor 4 binds to interleukin-8 receptors and activates neutrophils when its N terminus is modified with Glu-Leu-Arg. *Proc Natl Acad Sci USA* 1993; **90**: 3574–3577.
75. Gong J-H, Clark-Lewis I. Antagonists of monocyte chemoattractant protein-1 identified by modification of functionally critical NH2-terminal residues. *J Exp Med* 1995; **181**: 631–640.
76. Beall CJ, Mahajan S, Kolattukudy PE. Conversion of monocyte chemoattractant protein-1 into a neutrophil attractant by substitution of two amino acids. *J Biol Chem* 1992; **267**: 3455–3459.
77. Lasky LA. Combinatorial mediators of inflammation? *Curr Biol* 1993; **3**: 366–368.
78. Baggiolini M, Dahinden CA. CC chemokines in allergic inflammation. *Immunology Today* 1994; **15**: 127–133.
79. Springer TA. Traffic signals for lymphocyte recirculation and leukocyte emigration: the multistep paradigm. *Cell* 1994; **76**: 301–314.
80. Butcher EC Leukocyte-endothelial cell recognition: three (or more) steps to specificity and diversity. *Cell* 1991; **67**: 1033–1036.
81. Laskey LA. Selectins: interpreters of cell-specific carbohydrate information during inflammation. *Science* 1992; **258**: 964–969.
82. Shimizu Y, Newman W, Tanaka Y. S Lymphocyte interactions with endothelial cells. *Immunol Today* 1992; **13**: 106–112.
83. Roitt I, Brostoff J, Male D. *Immunology*. 3rd edition. St Louis: Mosby, 1993.
84. Devreotes PN, Zigmond SH. Chemotaxis in eukaryotic cells: a focus on leukocytes and *Dictyostelium*. *Annu Rev Cell Biol* 1988; **4**: 649–686.
85. Tanaka Y, Adams DH, Huscher S, Hirano H, Siebenlist U, Shaw S. T-cell adhesion induced by proteoglycan-immobilized cytokine MIP-1β. *Nature* 1993; **361**: 79–82.
86. Ebisawa M, Yamada T, Bickel C, Klunk D, Schleimer RP. Eosinophil transendothelial migration induced by cytokines. III. Effect of the chemokine RANTES. *J Immunol* 1994; **153**: 2153–2160.
87. Dahinden CA, Geiser T, Brunner T, Tscharner VV, Caput D, Ferrara P, Minty A, Baggiolini M. Monocyte chemotactic protein 3 is a most effective basophil- and eosinophil-activating chemokine. *J Exp Med* 1994; **179**: 751–756.
88. Ben-Baruch A, Xu L, Young PR, Bengali K, Oppenheim JJ, Wang JM. Monocyte chemotactic protein-3 (MCP-3) interacts with multiple leukocyte receptors. *J Biol Chem* 1995; **270**: 22123–22128.
89. Proost P, Wuyts A, Damme JV. Human monocyte chemotactic proteins-2 and -3: structural and functional comparison with MCP-1. *J Leuk Biol* 1996; **59**: 67–74.
90. Rothenberg ME, Luster AD, Leder P. Murine eotaxin: an eosinophil chemoattractant inducible in endothelial cells and in interleukin 4-induced tumor suppression. *Proc Natl Acad Sci USA* 1995; **92**: 8960–8964.
91. Griffiths-Johnson DA, Collins PD, Rossi AG, Jose PJ, Williams TJ. The chemokine eotaxin, activates guinea-pig eosinophils *in vitro* and causes their accumulation into the lung *in vivo*. *Biochem Biophys Res Comm* 1993; **197**: 1167–1172.
92. Rothenberg ME, Luster AD, Lilly CM, Drazen JM, Leder P. Constitutive and allergen-induced expression of eotaxin mRNA in the guinea pig lung. *J Exp Med* 1995; **181**: 1211–1216.
93. In: Sanver Techo, December 1995.
94. Ponath PD, Qin S, Ringler DJ, Clark-Lewis I, Wang J, Kassam N, Smith H, Shi X, Gonzalo J-A, Newman W, Gutierrez-Ramos J-C, Mackay CR. Cloning of the human eosinophil chemoattractant, eotaxin. *J Clin Invest* 1996; **97**: 604–612.
95. Stellato C, Beck LA, Gorgone GA, Proud D, Schall TJ, Ono SJ, Lichtenstein LM, Schleimer RP. Expression of the chemokine RANTES by a human bronchial epithelial cell line. Modulation by cytokines and glucocorticoids. *J Immunol* 1995; **155**: 410–418.
96. Rang HP, Dale MM, Ritter JM. Anti-inflammatory and immunosuppressant drugs. In: *Pharmacology*. New York: Churchill Livingstone, 1995.

ACKNOWLEDGEMENT. Dr E. Knol from the Centraal Laboratorium voor Bloedtransfusiedienst (CLB) is gratefully acknowledged for the valuable discussions during the preparation of the manuscript.

**Received 7 October 1996; accepted 8 October 1996**

## References

[PDF_01871] Adelroth E., Rosenhall L., Johansson S. A., Linden M., Venge P. (1990). Inflammatory cells and eosinophilic activity in asthmatics investigated by bronchoalveolar lavage. The effects of antiasthmatic treatment with budesonide or terbutaline.. Am Rev Respir Dis.

[PDF_01973] Ahuja S. K., Murphy P. M. (1993). Molecular piracy of mammalian interleukin-8 receptor type B by herpesvirus saimiri.. J Biol Chem.

[PDF_01883] Azzawi M., Bradley B., Jeffery P. K., Frew A. J., Wardlaw A. J., Knowles G., Assoufi B., Collins J. V., Durham S., Kay A. B. (1990). Identification of activated T lymphocytes and eosinophils in bronchial biopsies in stable atopic asthma.. Am Rev Respir Dis.

[PDF_01910] BOYDEN S. (1962). The chemotactic effect of mixtures of antibody and antigen on polymorphonuclear leucocytes.. J Exp Med.

[PDF_02092] Baggiolini M., Dahinden C. A. (1994). CC chemokines in allergic inflammation.. Immunol Today.

[PDF_01900] Baggiolini M., Dewald B., Moser B. (1994). Interleukin-8 and related chemotactic cytokines--CXC and CC chemokines.. Adv Immunol.

[PDF_01989] Baggiolini M., Loetscher P., Moser B. (1995). Interleukin-8 and the chemokine family.. Int J Immunopharmacol.

[PDF_01847] Barnes P. J. (1993). Anti-inflammatory therapy for asthma.. Annu Rev Med.

[PDF_02087] Beall C. J., Mahajan S., Kolattukudy P. E. (1992). Conversion of monocyte chemoattractant protein-1 into a neutrophil attractant by substitution of two amino acids.. J Biol Chem.

[PDF_01878] Beasley R., Roche W. R., Roberts J. A., Holgate S. T. (1989). Cellular events in the bronchi in mild asthma and after bronchial provocation.. Am Rev Respir Dis.

[PDF_02118] Ben-Baruch A., Xu L., Young P. R., Bengali K., Oppenheim J. J., Wang J. M. (1995). Monocyte chemotactic protein-3 (MCP3) interacts with multiple leukocyte receptors. C-C CKR1, a receptor for macrophage inflammatory protein-1 alpha/Rantes, is also a functional receptor for MCP3.. J Biol Chem.

[PDF_01998] Besemer J., Hujber A., Kuhn B. (1989). Specific binding, internalization, and degradation of human neutrophil activating factor by human polymorphonuclear leukocytes.. J Biol Chem.

[PDF_01887] Bochner B. S., Undem B. J., Lichtenstein L. M. (1994). Immunological aspects of allergic asthma.. Annu Rev Immunol.

[PDF_02096] Butcher E. C. (1991). Leukocyte-endothelial cell recognition: three (or more) steps to specificity and diversity.. Cell.

[PDF_02044] Charo I. F., Myers S. J., Herman A., Franci C., Connolly A. J., Coughlin S. R. (1994). Molecular cloning and functional expression of two monocyte chemoattractant protein 1 receptors reveals alternative splicing of the carboxyl-terminal tails.. Proc Natl Acad Sci U S A.

[PDF_01961] Chaudhuri A., Polyakova J., Zbrzezna V., Williams K., Gulati S., Pogo A. O. (1993). Cloning of glycoprotein D cDNA, which encodes the major subunit of the Duffy blood group system and the receptor for the Plasmodium vivax malaria parasite.. Proc Natl Acad Sci U S A.

[PDF_02004] Chuntharapai A., Kim K. J. (1995). Regulation of the expression of IL-8 receptor A/B by IL-8: possible functions of each receptor.. J Immunol.

[PDF_02007] Chuntharapai A., Lee J., Hébert C. A., Kim K. J. (1994). Monoclonal antibodies detect different distribution patterns of IL-8 receptor A and IL-8 receptor B on human peripheral blood leukocytes.. J Immunol.

[PDF_02080] Clark-Lewis I., Dewald B., Geiser T., Moser B., Baggiolini M. (1993). Platelet factor 4 binds to interleukin 8 receptors and activates neutrophils when its N terminus is modified with Glu-Leu-Arg.. Proc Natl Acad Sci U S A.

[PDF_01949] Clark-Lewis I., Kim K. S., Rajarathnam K., Gong J. H., Dewald B., Moser B., Baggiolini M., Sykes B. D. (1995). Structure-activity relationships of chemokines.. J Leukoc Biol.

[PDF_02020] Clark-Lewis I., Schumacher C., Baggiolini M., Moser B. (1991). Structure-activity relationships of interleukin-8 determined using chemically synthesized analogs. Critical role of NH2-terminal residues and evidence for uncoupling of neutrophil chemotaxis, exocytosis, and receptor binding activities.. J Biol Chem.

[PDF_02048] Combadiere C., Ahuja S. K., Murphy P. M. (1995). Cloning and functional expression of a human eosinophil CC chemokine receptor.. J Biol Chem.

[PDF_02051] Combadiere C., Ahuja S. K., Murphy P. M. (1995). Cloning, chromosomal localization, and RNA expression of a human beta chemokine receptor-like gene.. DNA Cell Biol.

[PDF_02114] Dahinden C. A., Geiser T., Brunner T., von Tscharner V., Caput D., Ferrara P., Minty A., Baggiolini M. (1994). Monocyte chemotactic protein 3 is a most effective basophil- and eosinophil-activating chemokine.. J Exp Med.

[PDF_01889] Dale H. H., Laidlaw P. P. (1910). The physiological action of beta-iminazolylethylamine.. J Physiol.

[PDF_02067] Darbonne W. C., Rice G. C., Mohler M. A., Apple T., Hébert C. A., Valente A. J., Baker J. B. (1991). Red blood cells are a sink for interleukin 8, a leukocyte chemotaxin.. J Clin Invest.

[PDF_02104] Devreotes P. N., Zigmond S. H. (1988). Chemotaxis in eukaryotic cells: a focus on leukocytes and Dictyostelium.. Annu Rev Cell Biol.

[PDF_01952] Farber J. M. (1993). HuMig: a new human member of the chemokine family of cytokines.. Biochem Biophys Res Commun.

[PDF_02031] Gao J. L., Kuhns D. B., Tiffany H. L., McDermott D., Li X., Francke U., Murphy P. M. (1993). Structure and functional expression of the human macrophage inflammatory protein 1 alpha/RANTES receptor.. J Exp Med.

[PDF_02084] Gong J. H., Clark-Lewis I. (1995). Antagonists of monocyte chemoattractant protein 1 identified by modification of functionally critical NH2-terminal residues.. J Exp Med.

[PDF_02128] Griffiths-Johnson D. A., Collins P. D., Rossi A. G., Jose P. J., Williams T. J. (1993). The chemokine, eotaxin, activates guinea-pig eosinophils in vitro and causes their accumulation into the lung in vivo.. Biochem Biophys Res Commun.

[PDF_02001] Grob P. M., David E., Warren T. C., DeLeon R. P., Farina P. R., Homon C. A. (1990). Characterization of a receptor for human monocyte-derived neutrophil chemotactic factor/interleukin-8.. J Biol Chem.

[PDF_01897] Gundel R. H., Letts L. G., Gleich G. J. (1991). Human eosinophil major basic protein induces airway constriction and airway hyperresponsiveness in primates.. J Clin Invest.

[PDF_01894] Henocq E., Vargaftig B. B. (1986). Accumulation of eosinophils in response to intracutaneous PAF-acether and allergens in man.. Lancet.

[PDF_02070] Holmes W. E., Lee J., Kuang W. J., Rice G. C., Wood W. I. (1991). Structure and functional expression of a human interleukin-8 receptor.. Science.

[PDF_01966] Horuk R., Colby T. J., Darbonne W. C., Schall T. J., Neote K. (1993). The human erythrocyte inflammatory peptide (chemokine) receptor. Biochemical characterization, solubilization, and development of a binding assay for the soluble receptor.. Biochemistry.

[PDF_01906] Horuk R. (1994). Molecular properties of the chemokine receptor family.. Trends Pharmacol Sci.

[PDF_02017] Hébert C. A., Chuntharapai A., Smith M., Colby T., Kim J., Horuk R. (1993). Partial functional mapping of the human interleukin-8 type A receptor. Identification of a major ligand binding domain.. J Biol Chem.

[PDF_02025] Hébert C. A., Vitangcol R. V., Baker J. B. (1991). Scanning mutagenesis of interleukin-8 identifies a cluster of residues required for receptor binding.. J Biol Chem.

[PDF_01875] Jeffery P. K., Wardlaw A. J., Nelson F. C., Collins J. V., Kay A. B. (1989). Bronchial biopsies in asthma. An ultrastructural, quantitative study and correlation with hyperreactivity.. Am Rev Respir Dis.

[PDF_02054] Jose P. J., Griffiths-Johnson D. A., Collins P. D., Walsh D. T., Moqbel R., Totty N. F., Truong O., Hsuan J. J., Williams T. J. (1994). Eotaxin: a potent eosinophil chemoattractant cytokine detected in a guinea pig model of allergic airways inflammation.. J Exp Med.

[PDF_01860] Kay A. B., Corrigan C. J. (1992). Asthma. Eosinophils and neutrophils.. Br Med Bull.

[PDF_01938] Kelner G. S., Kennedy J., Bacon K. B., Kleyensteuber S., Largaespada D. A., Jenkins N. A., Copeland N. G., Bazan J. F., Moore K. W., Schall T. J. (1994). Lymphotactin: a cytokine that represents a new class of chemokine.. Science.

[PDF_01942] Kelner G. S., Zlotnik A. (1995). Cytokine production profile of early thymocytes and the characterization of a new class of chemokine.. J Leukoc Biol.

[PDF_01991] Kelvin D. J., Michiel D. F., Johnston J. A., Lloyd A. R., Sprenger H., Oppenheim J. J., Wang J. M. (1993). Chemokines and serpentines: the molecular biology of chemokine receptors.. J Leukoc Biol.

[PDF_01945] Kennedy J., Kelner G. S., Kleyensteuber S., Schall T. J., Weiss M. C., Yssel H., Schneider P. V., Cocks B. G., Bacon K. B., Zlotnik A. (1995). Molecular cloning and functional characterization of human lymphotactin.. J Immunol.

[PDF_01865] Kirby J. G., Hargreave F. E., Gleich G. J., O'Byrne P. M. (1987). Bronchoalveolar cell profiles of asthmatic and nonasthmatic subjects.. Am Rev Respir Dis.

[PDF_02090] Lasky L. A. (1993). Chemokines: combinatorial mediators of inflammation?. Curr Biol.

[PDF_02098] Lasky L. A. (1992). Selectins: interpreters of cell-specific carbohydrate information during inflammation.. Science.

[PDF_02010] Lee J., Horuk R., Rice G. C., Bennett G. L., Camerato T., Wood W. I. (1992). Characterization of two high affinity human interleukin-8 receptors.. J Biol Chem.

[PDF_02013] Loetscher P., Seitz M., Clark-Lewis I., Baggiolini M., Moser B. (1994). Both interleukin-8 receptors independently mediate chemotaxis. Jurkat cells transfected with IL-8R1 or IL-8R2 migrate in response to IL-8, GRO alpha and NAP-2.. FEBS Lett.

[PDF_01918] Luster A. D., Unkeless J. C., Ravetch J. V. (1985). Gamma-interferon transcriptionally regulates an early-response gene containing homology to platelet proteins.. Nature.

[PDF_02073] Lusti-Narasimhan M., Power C. A., Allet B., Alouani S., Bacon K. B., Mermod J. J., Proudfoot A. E., Wells T. N. (1995). Mutation of Leu25 and Val27 introduces CC chemokine activity into interleukin-8.. J Biol Chem.

[PDF_01930] Meurer R., Van Riper G., Feeney W., Cunningham P., Hora D., Springer M. S., MacIntyre D. E., Rosen H. (1993). Formation of eosinophilic and monocytic intradermal inflammatory sites in the dog by injection of human RANTES but not human monocyte chemoattractant protein 1, human macrophage inflammatory protein 1 alpha, or human interleukin 8.. J Exp Med.

[PDF_02077] Moser B., Dewald B., Barella L., Schumacher C., Baggiolini M., Clark-Lewis I. (1993). Interleukin-8 antagonists generated by N-terminal modification.. J Biol Chem.

[PDF_01980] Moser B., Schumacher C., von Tscharner V., Clark-Lewis I., Baggiolini M. (1991). Neutrophil-activating peptide 2 and gro/melanoma growth-stimulatory activity interact with neutrophil-activating peptide 1/interleukin 8 receptors on human neutrophils.. J Biol Chem.

[PDF_01913] Murphy P. M. (1994). The molecular biology of leukocyte chemoattractant receptors.. Annu Rev Immunol.

[PDF_02064] Neote K., Darbonne W., Ogez J., Horuk R., Schall T. J. (1993). Identification of a promiscuous inflammatory peptide receptor on the surface of red blood cells.. J Biol Chem.

[PDF_01970] Neote K., DiGregorio D., Mak J. Y., Horuk R., Schall T. J. (1993). Molecular cloning, functional expression, and signaling characteristics of a C-C chemokine receptor.. Cell.

[PDF_01881] O'Byrne P. M. (1995). What is asthma? An update on the mechanisms.. J Investig Allergol Clin Immunol.

[PDF_01915] Oppenheim J. J., Zachariae C. O., Mukaida N., Matsushima K. (1991). Properties of the novel proinflammatory supergene "intercrine" cytokine family.. Annu Rev Immunol.

[PDF_02136] Ponath P. D., Qin S., Ringler D. J., Clark-Lewis I., Wang J., Kassam N., Smith H., Shi X., Gonzalo J. A., Newman W. (1996). Cloning of the human eosinophil chemoattractant, eotaxin. Expression, receptor binding, and functional properties suggest a mechanism for the selective recruitment of eosinophils.. J Clin Invest.

[PDF_02028] Probst W. C., Snyder L. A., Schuster D. I., Brosius J., Sealfon S. C. (1992). Sequence alignment of the G-protein coupled receptor superfamily.. DNA Cell Biol.

[PDF_01954] Proost P., De Wolf-Peeters C., Conings R., Opdenakker G., Billiau A., Van Damme J. (1993). Identification of a novel granulocyte chemotactic protein (GCP-2) from human tumor cells. In vitro and in vivo comparison with natural forms of GRO, IP-10, and IL-8.. J Immunol.

[PDF_02121] Proost P., Wuyts A., Van Damme J. (1996). Human monocyte chemotactic proteins-2 and -3: structural and functional comparison with MCP-1.. J Leukoc Biol.

[PDF_02058] Raport C. J., Schweickart V. L., Chantry D., Eddy R. L., Shows T. B., Godiska R., Gray P. W. (1996). New members of the chemokine receptor gene family.. J Leukoc Biol.

[PDF_02124] Rothenberg M. E., Luster A. D., Leder P. (1995). Murine eotaxin: an eosinophil chemoattractant inducible in endothelial cells and in interleukin 4-induced tumor suppression.. Proc Natl Acad Sci U S A.

[PDF_02132] Rothenberg M. E., Luster A. D., Lilly C. M., Drazen J. M., Leder P. (1995). Constitutive and allergen-induced expression of eotaxin mRNA in the guinea pig lung.. J Exp Med.

[PDF_01994] Samanta A. K., Oppenheim J. J., Matsushima K. (1990). Interleukin 8 (monocyte-derived neutrophil chemotactic factor) dynamically regulates its own receptor expression on human neutrophils.. J Biol Chem.

[PDF_01959] Schall T. J., Bacon K. B. (1994). Chemokines, leukocyte trafficking, and inflammation.. Curr Opin Immunol.

[PDF_01985] Schumacher C., Clark-Lewis I., Baggiolini M., Moser B. (1992). High- and low-affinity binding of GRO alpha and neutrophil-activating peptide 2 to interleukin 8 receptors on human neutrophils.. Proc Natl Acad Sci U S A.

[PDF_01852] Shelhamer J. H., Levine S. J., Wu T., Jacoby D. B., Kaliner M. A., Rennard S. I. (1995). NIH conference. Airway inflammation.. Ann Intern Med.

[PDF_02100] Shimizu Y., Newman W., Tanaka Y., Shaw S. (1992). Lymphocyte interactions with endothelial cells.. Immunol Today.

[PDF_02094] Springer T. A. (1994). Traffic signals for lymphocyte recirculation and leukocyte emigration: the multistep paradigm.. Cell.

[PDF_02140] Stellato C., Beck L. A., Gorgone G. A., Proud D., Schall T. J., Ono S. J., Lichtenstein L. M., Schleimer R. P. (1995). Expression of the chemokine RANTES by a human bronchial epithelial cell line. Modulation by cytokines and glucocorticoids.. J Immunol.

[PDF_01902] Strieter R. M., Koch A. E., Antony V. B., Fick R. B., Standiford T. J., Kunkel S. L. (1994). The immunopathology of chemotactic cytokines: the role of interleukin-8 and monocyte chemoattractant protein-1.. J Lab Clin Med.

[PDF_02106] Tanaka Y., Adams D. H., Hubscher S., Hirano H., Siebenlist U., Shaw S. (1993). T-cell adhesion induced by proteoglycan-immobilized cytokine MIP-1 beta.. Nature.

[PDF_01849] Virchow J. C., Kroegel C., Walker C., Matthys H. (1994). Cellular and immunological markers of allergic and intrinsic bronchial asthma.. Lung.

[PDF_02040] Wang J. M., McVicar D. W., Oppenheim J. J., Kelvin D. J. (1993). Identification of RANTES receptors on human monocytic cells: competition for binding and desensitization by homologous chemotactic cytokines.. J Exp Med.

[PDF_01868] Wardlaw A. J., Dunnette S., Gleich G. J., Collins J. V., Kay A. B. (1988). Eosinophils and mast cells in bronchoalveolar lavage in subjects with mild asthma. Relationship to bronchial hyperreactivity.. Am Rev Respir Dis.

[PDF_02061] Wells T. N., Power C. A., Lusti-Narasimhan M., Hoogewerf A. J., Cooke R. M., Chung C. W., Peitsch M. C., Proudfoot A. E. (1996). Selectivity and antagonism of chemokine receptors.. J Leukoc Biol.

[PDF_01926] Yoshimura T., Matsushima K., Oppenheim J. J., Leonard E. J. (1987). Neutrophil chemotactic factor produced by lipopolysaccharide (LPS)-stimulated human blood mononuclear leukocytes: partial characterization and separation from interleukin 1 (IL 1).. J Immunol.

[PDF_01921] Yoshimura T., Matsushima K., Tanaka S., Robinson E. A., Appella E., Oppenheim J. J., Leonard E. J. (1987). Purification of a human monocyte-derived neutrophil chemotactic factor that has peptide sequence similarity to other host defense cytokines.. Proc Natl Acad Sci U S A.

